# Supplementary material for: Innate cell markers that predict anti-HIV neutralizing antibody titers in vaccinated macaques
Source: Cell Rep Med. 2022 Sep 26;3(10):100751. doi: 10.1016/j.xcrm.2022.100751 (PMC9588994; doi:10.1016/j.xcrm.2022.100751)
Supplement: Document S1. Figures S1–S7 and Tables S1–S3 [file mmc1.pdf]

Cell Reports Medicine, Volume *3*

Supplemental information

Innate cell markers that predict anti-HIV neutralizing antibody titers in vaccinated macaques

Matthieu Van Tilbeurgh, Pauline Maisonnasse, Jean-Louis Palgen, Monica Tolazzi, Yoann Aldon, Nathalie Dereuddre-Bosquet, Mariangela Cavarelli, Anne-Sophie Beignon, Ernesto Marcos-Lopez, Anne-Sophie Gallouet, Emmanuel Gilson, Gabriel Ozorowski, Andrew B. Ward, Ilja Bontjer, Paul F. McKay, Robin J. Shattock, Gabriella Scarlatti, Rogier W. Sanders, and Roger Le Grand

Innate cell markers that predict anti-HIV neutralizing antibody titers in vaccinated macaques

Supplementary materials

Authors: Matthieu Van Tilbeurgh[1], Pauline Maisonnasse[1], Jean-Louis Palgen[1], Monica Tolazzi[2], Yoann Aldon[3], Nathalie Dereuddre-Bosquet[1], Mariangela Cavarelli[1], Anne-Sophie Beignon[1], Ernesto Marcos-Lopez[1], Anne-Sophie Gallouet[1], Emmanuel Gilson[4], Gabriel Ozorowski[5], Andrew B. Ward[5], Ilja Bontjer[6], Paul F. McKay[3], Robin J. Shattock[3], Gabriella Scarlatti[2], Rogier W. Sanders[6,7], and Roger Le Grand[1]*.

Affiliations:

[1] Université Paris-Saclay, Inserm, CEA, Center for Immunology of Viral, Auto-immune, Hematological and Bacterial diseases » (IMVA-HB/IDMIT), 92265 Fontenay-aux-Roses & 94270 Le Kremlin-Bicêtre, France

[2] Viral Evolution and Transmission Unit, Division of Immunology, Transplantation and Infectious Diseases, IRCCS Ospedale San Raffaele, 20132 Milan, Italy

[3] Imperial College London, Faculty of Medicine, Department of Infectious Disease, London, United Kingdom.

[4] Life & Soft, 28 rue de la Redoute, 92260 Fontenay-aux-Roses, France

[5] Department of Integrative Structural and Computational Biology, The Scripps Research Institute, La Jolla, CA 92037, USA.

[6] Department of Medical Microbiology and Infection Prevention, Amsterdam University Medical Centers, Location AMC, University of Amsterdam, 1105 AZ Amsterdam, the Netherlands

[7] Department of Microbiology and Immunology, Weill Medical College of Cornell University, New York, NY 10021, USA

* Corresponding author: roger.le-grand@cea.fr

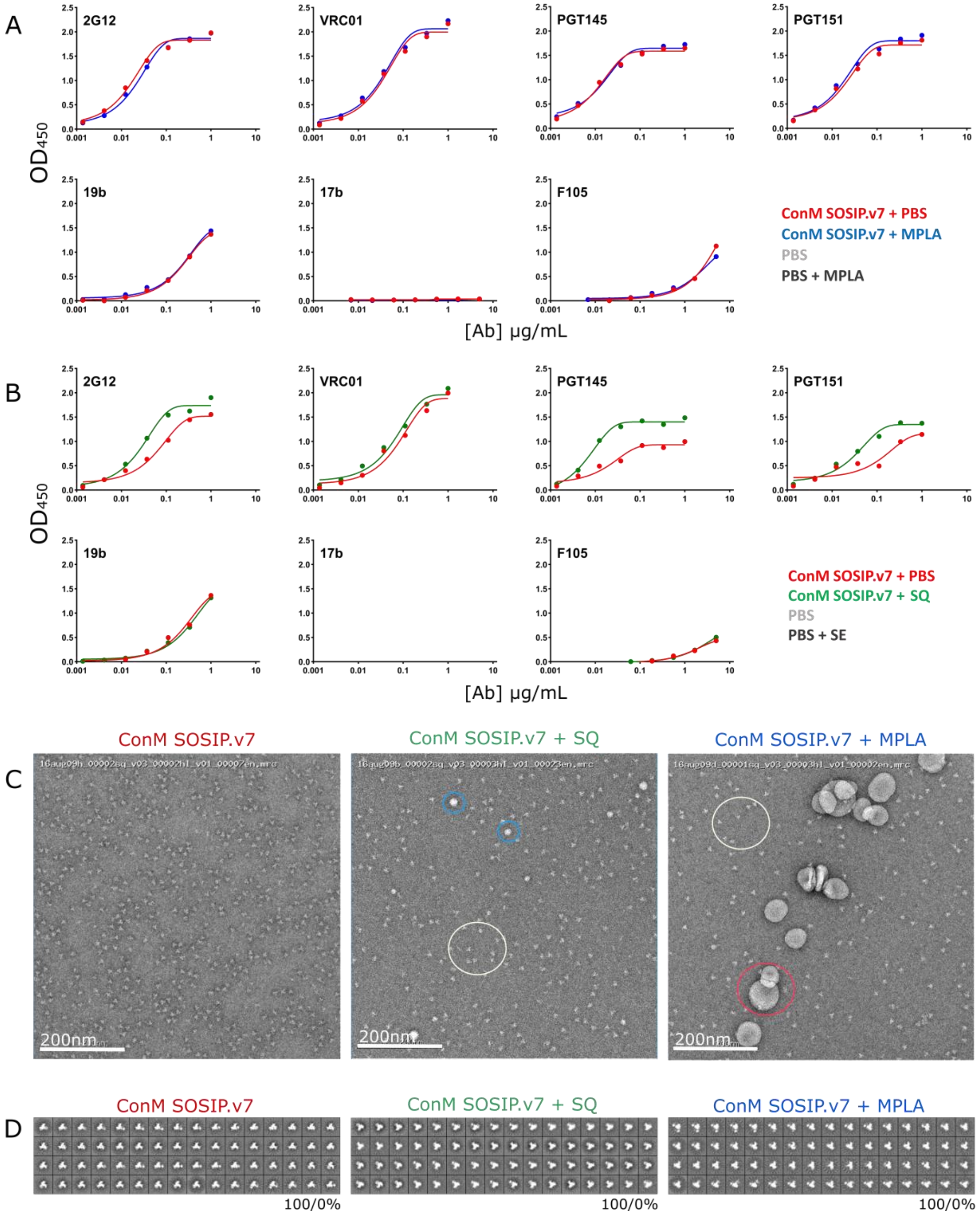

Figure S1. Formulation of ConM SOSIP.v7 with MPLA and SQ adjuvants (related to figure 2). D7324-tagged ConM SOSIP.v7 was incubated with the corresponding adjuvant for 1 h at 37ºC and the antigenicity (**A**, **B**) and morphology (**C**, **D**) were subsequently assessed by D7324-capture ELISA and negative-stain electron microscopy, respectively. (**C**) Electron micrographs of ConM SOSIP.v7 plus adjuvants; White, blue and red circles identified respectively trimers, Squalene and MPLA. (**D**) 2D-class averages of ConM SOSIP.v7 proteins in formulations. Percentage of native-like/non-native trimers (see *Materials and Methods*) are listed below each set of class averages.

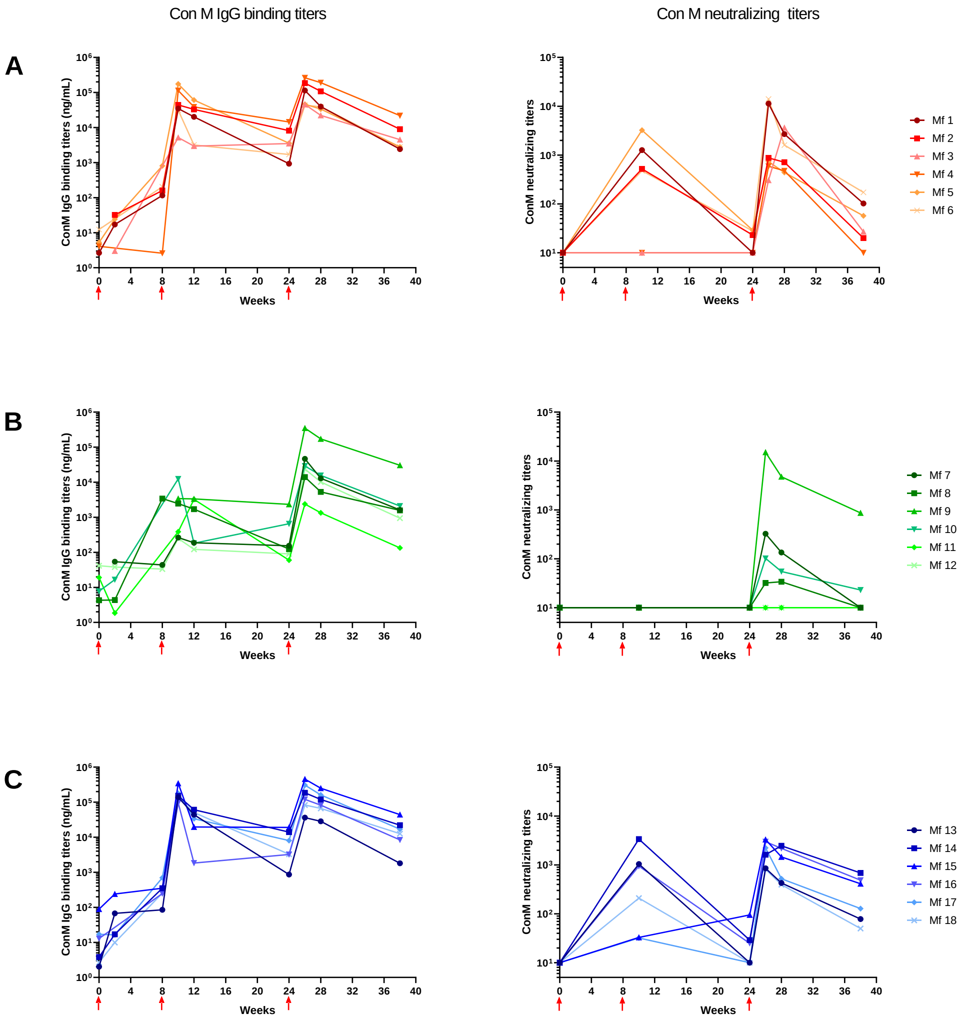

Figure S2. Individual ConM SOSIP v7 humoral responses (related to figure 2). ConM IgG binding and neutralizing titers of cynomolgus macaques from the MPLA-IM (A), MPLA-SC (B), and SQ-IM (C) groups, with red, green, and blue shading, respectively. Immunizations (week 0, 8, and 24) are indicated by the red arrows.

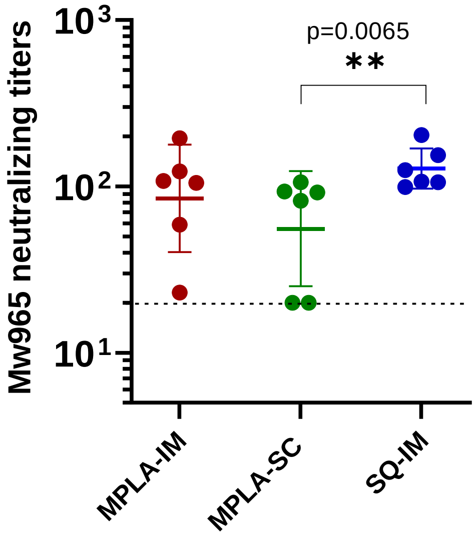

Figure S3: Heterologous neutralization against MW965 virus (related to figure 2). MW965 neutralizing titers were measured at week (W)28. Animals from the MPLA-IM group are displayed in red, the MPLA-SC group in green, and the SQ-IM group in blue. The geometric mean and geometric SD are represented. Comparison realized with a Mann-Whitney-Wilcoxon test with p.values < 0.05 are displayed.

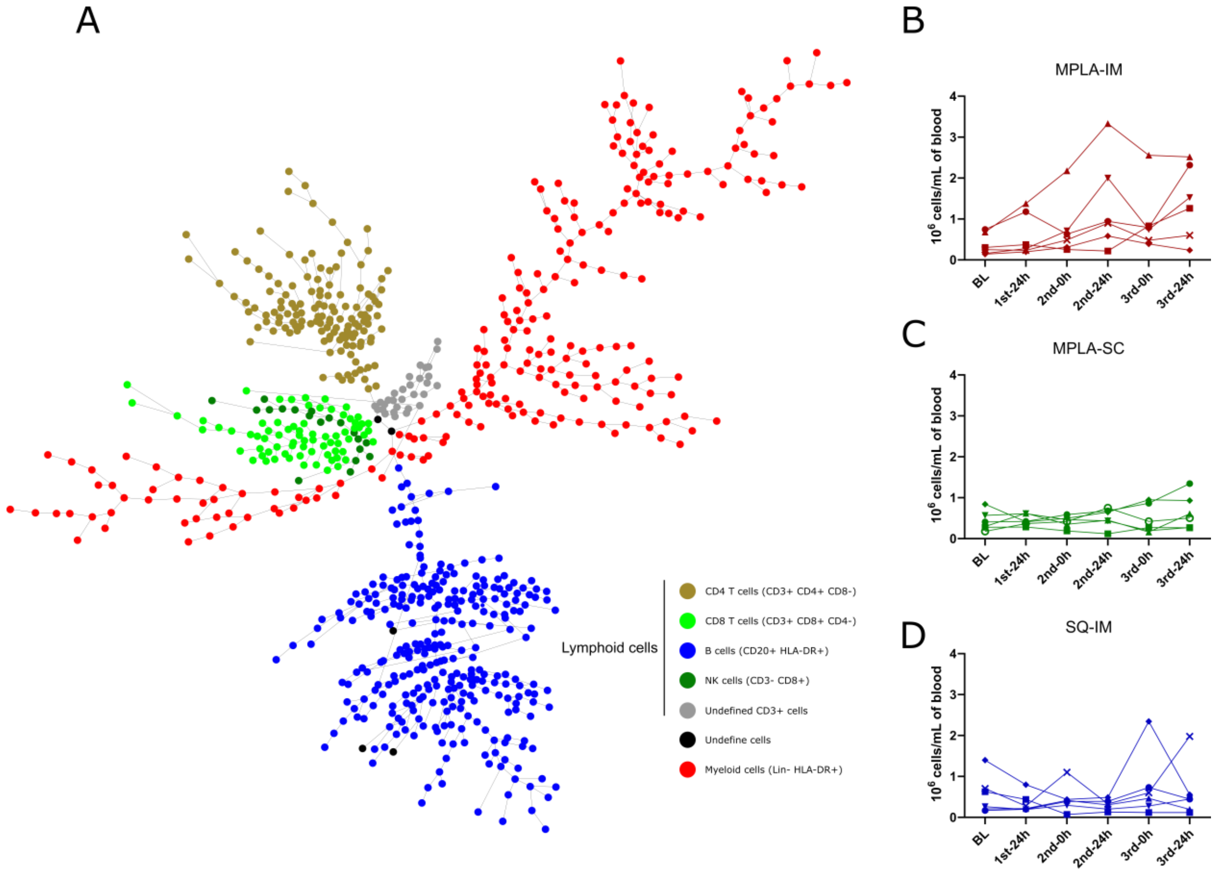

Figure S4. Identification of major cell populations using the SPADE clustering method following immunizations with SOSIP v7 ConM (related to figure 3). (**A**) SPADE tree created using the entire mass cytometry data set. Each dot represents a cell cluster. Major cell populations are annotated according to their phenotype: B cells ($CD20^{+}$ $HLA\text{-}DR^{+}$ $CD1c^{+}$), T cells ($CD3^{+}$ $CD4^{+}$ or $CD8^{+}$), NK cells ($CD3^{-}$ $CD8^{+}$), undefined lymphocytes ($CD3^{+}$ $CD4^{-}$ $CD8^{low/-}$), and undefined cells. All remaining cell clusters are considered to be myeloid cells. Phenotypically similar clusters were linked using a minimal spanning-tree approach of the SPADE algorithm. Kinetics of the myeloid cells compartment depending on the vaccine: (**B**) MPLA-IM, (**C**) MPLA-SC and (**D**) SQ-IM.

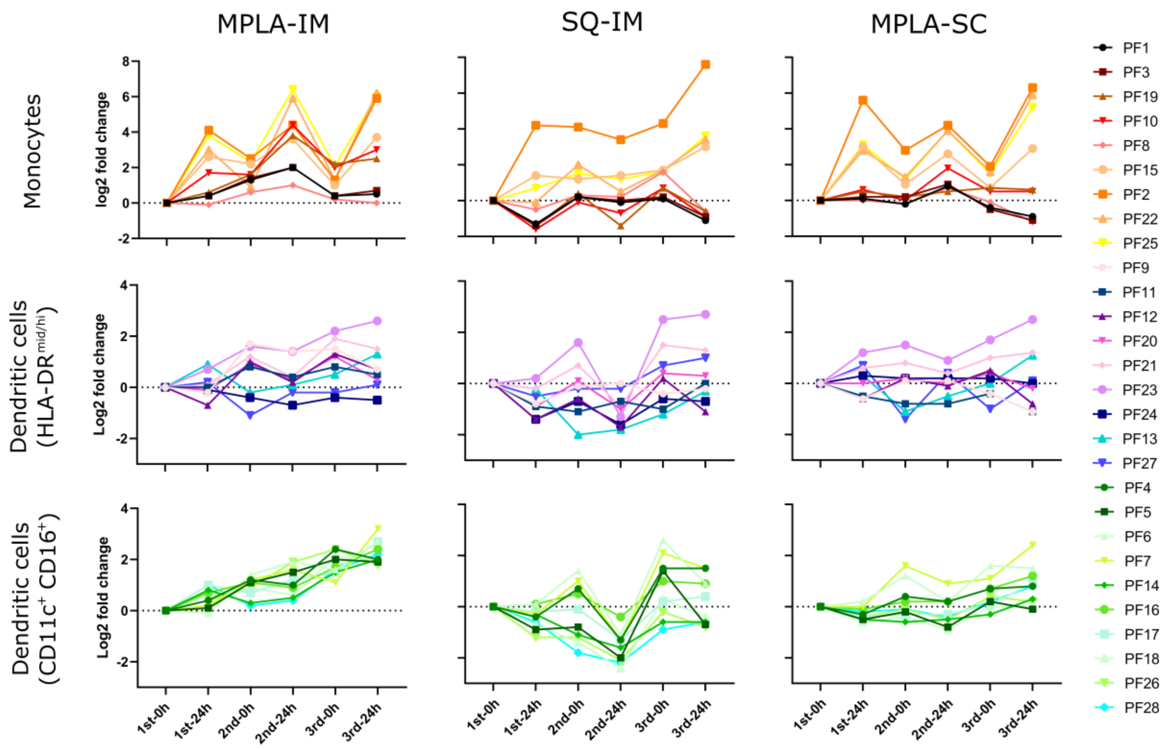

Figure S5. Variation of phenotypic families following immunization (related to figure 4). Mean log2 fold changes relative to the baseline values are displayed for each superfamily. Each color is assigned to a phenotypic family according to the colors defined in the heatmap (Fig.3).

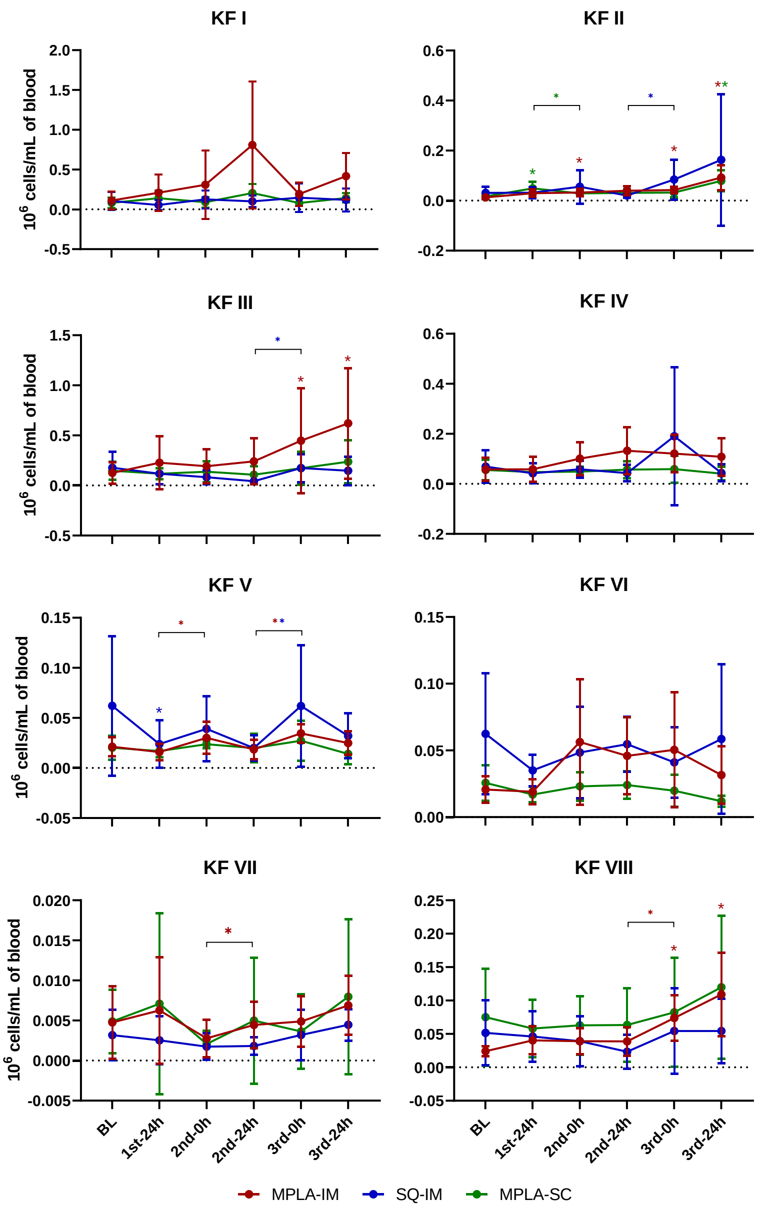

Figure S6. Dynamics of kinetic families following vaccine injections (related to figure 5). Phenotypic families were regrouped by similar dynamics to form eight kinetic families. The graphs show the mean cell number per mL of blood for all individuals (n=6) for each injection (MPLA-IM in red, MPLA-SC in green, and SQ-IM in blue). Significant differences relative to baseline values are indicated by a star and black lines are used to show significant differences compared to the previous time-point. P-values are shown in Table S2.

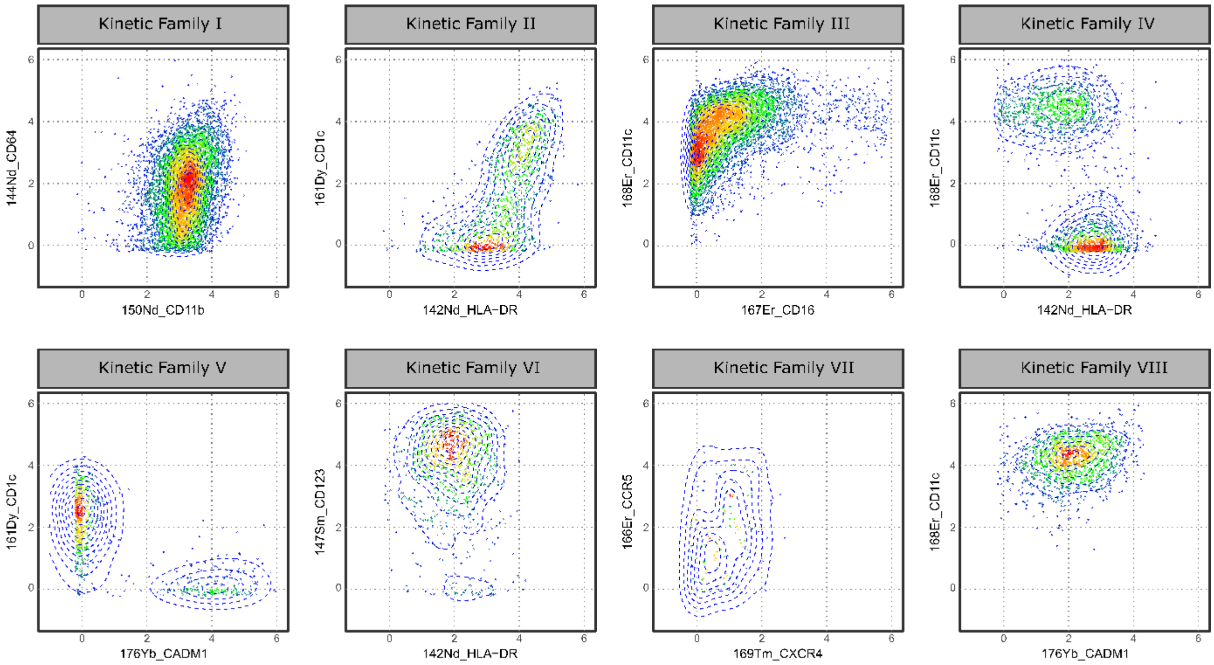

Figure S7: Main characteristics of kinetic families expression profiles (related to figure 5). Each dotplot represents the mean signal intensity (MSI) of all cells contained by a kinetic family (KF) without vaccines segregation. Markers are chosen according to the main population characteristics as defined in Table 1.

Table S1. **p-values of differences in cell numbers within phenotypic families (related to figure 4).** Comparisons were made using the Mann-Whitney Wilcoxon test. P-values ≤ 0.05 are shown in red.

	Conditions	Timepoints	PF1	PF3	PF19	PF10	PF8	PF15	PF2	PF22	PF25	PF12	PF23	PF21	PF20	PF9	PF24	PF11	PF27	PF13	PF28	PF17	PF5	PF4	PF26	PF16	PF14	PF6	PF18	PF7
Comparison between conditions	MPLA-SC vs SQ-IM	Baseline	0.94	0.70	0.59	0.82	0.82	0.82	0.70	0.94	0.82	0.09	0.18	0.18	0.09	0.48	0.39	**0.04**	0.31	0.18	0.94	0.82	1.00	0.70	0.48	0.39	1.00	0.94	0.70	0.82
		1st-24h	0.18	0.13	0.24	**0.04**	0.18	0.13	0.09	0.06	**0.03**	**0.03**	0.06	1.00	0.94	0.70	0.94	0.94	0.48	0.24	0.70	0.82	0.48	0.82	0.59	0.48	0.59	0.94	1.00	0.59
		2nd-0h	0.94	0.94	1.00	0.82	0.18	0.39	0.82	0.70	0.70	0.18	0.39	0.94	0.48	0.31	0.94	0.48	0.75	1.00	0.82	0.48	0.39	0.48	0.31	0.59	0.70	0.18	0.94	0.31
		2nd-24h	0.48	0.18	0.24	**0.02**	0.70	0.18	0.13	0.06	**0.02**	**0.03**	0.09	0.94	0.94	0.39	0.31	0.94	0.24	0.70	0.82	0.31	0.18	0.31	0.24	0.18	0.13	0.39	0.94	0.06
		3rd-0h	0.94	0.59	0.39	0.70	0.94	0.48	0.48	0.94	1.00	0.09	0.31	0.48	0.31	0.31	0.13	0.13	0.70	0.18	0.59	0.31	0.70	1.00	0.39	0.82	0.48	0.39	0.24	1.00
		3rd-24h	0.82	1.00	0.18	0.13	0.48	0.82	0.59	**0.03**	0.09	**0.00**	**0.002**	**0.03**	0.31	0.48	0.70	0.31	0.39	0.94	1.00	0.82	0.24	0.24	0.39	0.39	0.48	0.94	0.82	0.39
	SQ-IM vs MPLA-IM	Baseline	1.00	0.59	0.13	0.94	0.59	0.94	1.00	0.48	0.94	**0.03**	0.06	0.06	**0.03**	0.31	0.13	0.48	0.82	0.39	0.24	0.31	0.94	0.82	0.59	0.18	0.94	0.59	0.48	0.94
		1st-24h	0.18	0.06	0.24	**0.02**	0.18	0.06	0.24	**0.009**	**0.009**	**0.03**	0.31	0.39	0.24	0.13	0.09	0.48	0.18	0.13	0.94	0.48	1.00	0.94	0.94	0.94	0.48	0.94	1.00	0.82
		2nd-0h	0.48	0.39	0.82	0.39	0.39	1.00	0.82	0.94	0.70	0.94	1.00	1.00	0.94	0.31	0.31	0.59	0.13	0.70	0.59	0.31	0.31	0.70	0.82	0.82	0.24	0.24	0.48	0.24
		2nd-24h	0.09	**0.03**	**0.04**	0.06	0.06	**0.04**	**0.03**	0.06	**0.04**	0.59	0.48	1.00	0.70	0.82	0.59	1.00	0.06	0.09	0.31	0.09	**0.04**	0.39	0.24	0.18	0.09	0.31	0.31	0.13
		3rd-0h	0.06	0.06	0.06	**0.03**	0.09	0.18	0.59	0.06	0.09	0.39	0.59	0.59	0.48	0.48	0.94	0.82	0.09	0.59	0.59	0.13	**0.04**	0.09	0.13	0.24	0.06	0.31	0.24	0.09
		3rd-24h	0.39	0.31	1.00	0.13	0.31	0.94	0.59	0.59	0.59	0.94	0.48	0.94	0.70	0.48	**0.03**	0.39	0.18	1.00	0.70	1.00	1.00	0.94	0.18	0.59	0.09	0.70	0.94	0.09
	MPLA-IM vs MPLA-SC	Baseline	0.70	0.94	0.39	0.24	0.59	0.59	0.82	0.94	0.94	0.48	0.94	0.70	0.70	0.48	0.70	0.24	0.48	0.94	0.24	0.39	0.94	0.82	0.09	0.13	1.00	0.39	0.39	0.59
		1st-24h	0.82	0.94	0.94	0.48	1.00	1.00	0.59	0.48	0.82	0.94	0.94	0.13	0.31	**0.03**	0.09	0.70	0.48	1.00	0.70	0.70	0.48	0.94	0.39	0.48	0.70	0.59	0.94	1.00
		2nd-0h	0.31	0.13	0.94	0.39	**0.02**	0.24	0.59	0.48	0.39	0.18	0.18	0.94	0.59	0.82	0.24	0.39	0.59	0.59	0.82	0.94	0.70	0.70	0.24	0.82	0.48	0.94	0.39	0.94
		2nd-24h	0.18	0.31	0.31	0.09	0.09	0.06	0.18	0.09	0.09	0.13	0.59	0.82	0.94	0.39	0.82	0.70	0.48	0.31	0.59	0.59	0.39	0.82	0.82	0.59	0.39	0.70	0.59	0.48
		3rd-0h	0.24	**0.00**	0.09	0.09	**0.04**	0.13	1.00	0.24	0.13	0.06	0.59	0.70	0.31	0.59	0.94	0.06	0.48	0.18	0.48	0.13	0.18	0.48	0.48	0.82	0.18	0.18	0.24	0.31
		3rd-24h	**0.04**	0.06	0.39	0.09	**0.04**	0.06	0.59	0.94	0.82	0.31	0.13	0.06	0.48	0.82	0.70	0.70	0.70	0.39	0.31	0.18	0.70	0.31	0.82	0.94	0.18	0.31	0.24	0.24
Comparison with the previous timepoint	MPLA-IM	2nd-0h vs 1st-24h	0.22	0.09	0.31	0.84	0.09	0.22	0.06	0.09	0.06	**0.03**	0.31	**0.03**	**0.03**	**0.03**	0.09	0.31	0.31	0.06	0.09	0.16	**0.03**	**0.03**	0.84	0.22	1.00	0.44	0.22	0.56
		2nd-24h vs 2nd-0h	**0.03**	**0.03**	0.06	0.06	0.09	**0.03**	**0.03**	0.06	**0.03**	0.31	0.31	**0.03**	0.16	0.09	0.69	0.69	0.06	**0.03**	0.69	0.84	0.84	1.00	0.22	0.44	0.44	**0.03**	0.31	0.16
		3rd-0h vs 2nd-24h	**0.03**	**0.03**	0.22	0.06	0.22	0.06	0.06	0.06	0.06	0.69	0.69	**0.03**	0.09	0.06	0.31	1.00	0.69	1.00	**0.03**	0.22	1.00	0.69	0.06	0.16	0.44	**0.03**	0.31	**0.03**
		3rd-24h vs 3rd-0h	1.00	0.44	1.00	1.00	0.69	**0.03**	**0.03**	**0.03**	**0.03**	0.31	0.84	0.06	0.06	0.56	0.44	0.69	0.06	0.31	0.56	0.31	0.84	0.06	0.16	0.31	0.06	0.44	0.84	0.16
	MPLA-SC	2nd-0h vs 1st-24h	1.00	0.84	0.84	0.31	0.69	**0.03**	**0.03**	0.44	0.09	0.16	0.56	**0.03**	0.44	0.84	0.31	0.69	0.44	0.09	0.16	0.16	0.09	**0.03**	0.84	0.22	0.56	0.84	0.84	0.84
		2nd-24h vs 2nd-0h	0.16	0.16	0.84	**0.03**	0.16	**0.03**	0.16	**0.03**	**0.03**	0.69	1.00	0.56	0.56	0.31	0.22	1.00	0.31	0.06	0.44	0.22	0.22	0.22	1.00	0.84	1.00	0.16	0.44	0.31
		3rd-0h vs 2nd-24h	0.09	0.06	0.84	0.16	0.09	**0.03**	0.06	0.06	**0.03**	0.56	0.84	0.44	0.84	0.44	0.31	1.00	0.44	0.31	0.09	0.09	0.16	0.22	0.22	0.44	0.44	0.09	0.31	0.31
		3rd-24h vs 3rd-0h	0.22	0.44	0.84	1.00	0.09	**0.03**	**0.03**	**0.03**	**0.03**	**0.03**	1.00	0.09	0.84	1.00	0.31	1.00	0.09	0.16	1.00	0.69	1.00	**0.03**	0.16	0.16	0.06	0.84	0.56	0.06
	SQ-IM	2nd-0h vs 1st-24h	0.09	0.06	0.06	0.06	0.44	0.56	0.84	**0.03**	0.44	0.44	1.00	0.44	0.44	0.44	0.44	0.69	0.06	0.84	0.84	0.84	0.56	0.69	0.56	0.84	0.84	0.56	0.56	0.69
		2nd-24h vs 2nd-0h	0.84	0.84	0.16	0.31	0.69	0.84	0.56	0.44	0.56	0.56	0.09	0.06	0.16	**0.03**	**0.03**	0.22	0.44	0.84	0.06	0.16	0.16	0.16	0.31	0.06	0.06	0.16	0.44	0.22
		3rd-0h vs 2nd-24h	1.00	0.84	0.06	0.06	0.84	1.00	1.00	0.44	0.44	0.31	0.44	**0.03**	0.06	0.03	**0.03**	0.16	0.56	1.00	**0.03**	**0.03**	**0.03**	0.09	0.09	0.06	0.06	**0.03**	**0.03**	0.06
		3rd-24h vs 3rd-0h	0.09	1.00	0.16	**0.03**	0.06	0.31	**0.03**	0.31	0.22	0.69	0.31	0.16	0.69	0.84	0.44	0.69	0.22	0.69	1.00	0.16	0.44	1.00	1.00	0.84	0.69	0.56	0.44	1.00
Comparison with baseline	MPLA-IM	1st-24h	0.44	0.44	**0.03**	**0.03**	0.84	**0.03**	**0.03**	**0.03**	**0.03**	0.31	1.00	0.06	0.56	1.00	0.06	1.00	0.44	0.69	**0.03**	0.69	0.84	0.56	0.44	**0.03**	**0.03**	0.84	0.44	0.44
		2nd-0h	0.16	0.09	0.16	0.16	0.22	0.16	**0.03**	0.31	0.09	0.06	0.44	0.09	0.09	**0.03**	**0.03**	0.16	0.22	0.06	0.03	0.22	0.06	0.06	0.69	**0.03**	0.22	0.09	0.44	1.00
		2nd-24h	0.06	0.06	0.06	**0.03**	0.09	**0.03**	**0.03**	0.06	**0.03**	0.06	0.84	1.00	0.69	0.56	0.06	0.22	0.84	0.84	0.09	0.44	0.06	0.22	0.56	0.16	0.16	0.69	0.31	0.69
		3rd-0h	0.09	0.22	0.09	**0.03**	0.84	**0.03**	**0.03**	0.22	**0.03**	0.16	1.00	0.03	**0.03**	**0.03**	**0.03**	0.31	0.44	0.56	**0.03**	0.06	**0.03**	0.16	**0.03**	**0.03**	**0.03**	**0.03**	**0.03**	**0.03**
		3rd-24h	0.22	0.22	0.06	**0.03**	0.84	**0.03**	**0.03**	**0.03**	**0.03**	0.56	0.22	0.44	0.56	0.16	0.06	0.44	**0.03**	0.84	**0.03**	**0.03**	**0.03**	**0.03**	**0.03**	**0.03**	**0.03**	**0.03**	**0.03**	**0.03**
	MPLA-SC	1st-24h	1.00	1.00	0.31	0.31	1.00	**0.03**	**0.03**	0.06	**0.03**	0.31	0.56	0.56	1.00	0.22	**0.03**	0.84	1.00	0.84	0.44	0.44	0.16	1.00	0.56	1.00	1.00	0.31	0.31	0.84
		2nd-0h	0.56	0.84	0.84	0.84	0.56	0.16	0.06	0.09	0.16	0.84	0.31	0.44	0.69	0.09	0.16	0.84	0.31	**0.03**	0.44	0.84	0.09	**0.03**	0.56	0.69	1.00	0.56	0.56	0.44
		2nd-24h	0.44	0.16	0.69	**0.03**	0.16	**0.03**	**0.03**	**0.03**	**0.03**	1.00	0.56	1.00	1.00	0.44	0.06	0.44	0.09	0.44	0.44	0.31	0.44	0.22	0.16	1.00	0.84	0.22	0.44	0.56
		3rd-0h	0.84	0.84	0.31	0.44	0.84	0.09	**0.03**	**0.03**	0.06	0.69	0.44	0.16	0.84	0.44	0.06	1.00	0.84	0.06	1.00	0.56	0.44	0.16	0.44	0.56	1.00	1.00	0.69	0.84
		3rd-24h	0.22	0.31	0.44	0.31	0.16	**0.03**	**0.03**	**0.03**	**0.03**	0.16	**0.03**	0.16	0.56	0.22	**0.03**	1.00	0.31	0.44	0.69	1.00	0.09	**0.03**	0.84	0.09	0.16	1.00	1.00	0.31
	SQ-IM	1st-24h	**0.03**	**0.03**	**0.03**	0.06	0.06	0.09	**0.03**	0.84	0.22	0.22	0.16	0.03	0.16	0.44	0.84	**0.03**	0.56	0.31	0.03	0.16	1.00	0.69	0.31	0.56	0.31	0.16	0.06	0.06
		2nd-0h	0.84	0.69	0.69	1.00	0.56	0.22	0.06	0.06	0.16	0.84	0.16	0.69	0.69	0.44	0.31	1.00	0.16	0.56	1.00	0.69	0.31	0.44	0.22	0.84	1.00	0.56	0.09	0.16
		2nd-24h	0.84	0.84	0.16	0.44	0.31	0.03	**0.03**	0.69	0.69	1.00	0.31	0.09	0.16	0.22	0.09	**0.03**	0.16	0.56	0.06	**0.03**	0.09	0.22	0.22	0.56	0.09	**0.03**	0.06	0.06
		3rd-0h	1.00	0.84	0.31	0.16	0.84	0.09	0.06	0.09	0.06	0.31	0.06	0.44	0.31	**0.03**	**0.03**	0.56	0.69	0.84	0.22	0.22	0.06	0.22	0.31	0.44	0.56	0.31	1.00	0.44
		3rd-24h	0.09	0.31	0.69	0.69	0.31	**0.03**	**0.03**	0.06	**0.03**	1.00	0.84	0.22	1.00	0.16	0.09	0.44	0.84	0.16	0.31	1.00	0.44	0.31	1.00	0.22	0.56	1.00	0.84	0.84

Table S2. p-values of differences in cell number between kinetic families (related to figure 5). Comparisons were made using the Mann-Whitney Wilcoxon test. P-values ≤ 0.05 are shown in red.

	Conditions	Timepoint	KF I	KF II	KF III	KF IV	KF V	KF VI	KF VII	KF VIII
Comparison between conditions	MPLA-SC vs SQ-IM	Baseline	0.937	0.240	0.937	0.937	0.132	0.132	0.180	0.589
		1st-24h	**0.041**	0.394	0.485	0.132	0.937	**0.009**	0.485	0.394
		2nd-0h	0.699	0.589	0.240	0.589	0.485	0.065	0.589	0.394
		2nd-24h	0.065	0.132	0.240	0.699	0.937	**0.026**	0.699	0.240
		3rd-0h	0.937	0.180	0.485	0.485	0.394	0.132	0.589	0.485
		3rd-24h	0.310	0.818	0.394	0.937	0.132	**0.004**	0.589	0.240
	MPLA-IM vs SQ-IM	Baseline	0.937	0.240	0.699	0.937	0.132	**0.015**	0.699	0.394
		1st-24h	**0.041**	1.000	0.937	0.485	1.000	**0.041**	0.093	0.699
		2nd-0h	0.589	0.699	0.485	0.310	0.818	0.937	0.394	0.699
		2nd-24h	**0.026**	0.065	0.132	**0.041**	1.000	0.485	0.065	0.180
		3rd-0h	0.485	0.310	0.394	0.699	0.818	1.000	0.394	0.310
		3rd-24h	0.065	0.699	0.065	0.093	0.818	0.589	0.180	0.180
	MPLA-IM vs MPLA-SC	Baseline	0.818	0.485	0.699	1.000	0.589	0.485	0.818	0.132
		1st-24h	0.937	0.240	1.000	0.589	0.699	0.699	0.589	0.589
		2nd-0h	0.132	0.937	0.818	0.093	0.937	0.093	0.937	0.394
		2nd-24h	0.093	0.240	0.485	0.132	0.818	0.132	0.310	0.699
		3rd-0h	**0.041**	0.394	0.310	0.093	0.818	0.132	0.240	0.937
		3rd-24h	0.132	0.699	0.310	0.093	0.132	0.240	0.589	1.000
Comparison with the previous timepoint	MPLA-IM	2nd-0h vs 1st-24h	0.313	0.688	1.000	0.156	**0.031**	0.063	0.156	1.000
		2nd-24h vs 2nd-0h	**0.031**	0.438	0.219	0.156	0.063	0.313	**0.031**	1.000
		3rd-0h vs 2nd-24h	0.063	0.563	0.156	0.688	**0.031**	1.000	0.844	**0.031**
		3rd-24h vs 3rd-0h	0.063	0.063	0.563	0.219	0.063	0.563	0.063	0.219
	MPLA-SC	2nd-0h vs 1st-24h	0.219	**0.031**	0.844	0.844	0.094	0.094	0.219	0.313
		2nd-24h vs 2nd-0h	**0.031**	0.844	0.219	0.438	0.688	1.000	0.313	1.000
		3rd-0h vs 2nd-24h	0.063	0.688	0.313	1.000	0.313	0.563	0.688	0.313
		3rd-24h vs 3rd-0h	0.063	0.063	0.094	0.313	0.094	0.063	0.156	0.094
	SQ-IM	2nd-0h vs 1st-24h	0.156	0.438	1.000	0.563	0.438	0.563	0.688	0.563
		2nd-24h vs 2nd-0h	1.000	0.063	0.156	0.438	0.063	0.438	0.688	0.094
		3rd-0h vs 2nd-24h	0.844	**0.031**	**0.031**	0.063	**0.031**	0.438	0.563	0.063
		3rd-24h vs 3rd-0h	0.563	0.844	0.688	0.156	0.563	1.000	0.313	0.844
Comparison with baseline	MPLA-IM	1st-24h	**0.031**	0.063	0.313	0.844	0.219	0.219	0.438	0.156
		2nd-0h	0.156	**0.031**	0.219	0.219	0.688	0.094	0.063	0.156
		2nd-24h	**0.031**	0.063	0.219	0.156	0.438	0.156	0.844	0.313
		3rd-0h	0.063	**0.031**	**0.031**	0.156	0.063	0.156	0.844	**0.031**
		3rd-24h	**0.031**	**0.031**	**0.031**	0.438	0.688	0.438	0.219	**0.031**
	MPLA-SC	1st-24h	0.219	**0.031**	0.688	0.844	0.844	0.313	1.000	1.000
		2nd-0h	1.000	0.156	0.688	0.844	0.563	1.000	0.094	1.000
		2nd-24h	0.094	0.156	0.438	0.844	0.844	1.000	0.563	0.563
		3rd-0h	0.688	0.438	0.844	0.844	0.313	0.688	0.313	0.844
		3rd-24h	0.219	**0.031**	0.313	0.563	0.219	0.063	0.438	0.156
	SQ-IM	1st-24h	0.063	0.563	0.063	0.063	**0.031**	0.219	0.313	0.438
		2nd-0h	0.688	0.438	0.563	1.000	0.844	0.313	0.438	0.563
		2nd-24h	1.000	0.563	0.063	0.313	0.063	0.844	0.313	0.219
		3rd-0h	0.313	0.063	0.688	0.219	0.844	0.156	1.000	0.844
		3rd-24h	1.000	0.063	1.000	0.688	0.438	1.000	0.438	0.844

Table S3. Myeloid panel used for the mass-cytometry experiment (related to STAR Methods section). The metal and antibody clone associations are shown. Intracellular markers are shown in bold.

Metal	Antibody	Clone
Pr(141)	CD66abce	TET2
Nd(142)	HLA-DR	L243
Nd(143)	CD3	SP34.2
Nd(144)	CD64	10.1
Nd(145)	CD8	RPAT8
**Nd(146)**	**IL6**	**MQ2.13A5**
Sm(147)	CD123	7G3
**Nd(148)**	**IL4**	**8D48**
Sm(149)	CD11a	HI111
Nd(150)	CD11b	ICRF144
Eu(151)	CD62L	SK11
Sm(152)	CD4	L200
Eu(153)	FceRI	AER37
Sm(154)	CD86	IT2.2
Gd(156)	CD172a	15-414 / REA144
**Gd(158)**	**IP10**	**6D4**
Tb(159)	CD45	D058-1283
**Gd(160)**	**IL1a**	**364/3B3**
Dy(161)	CD1c	AF-5910
**Dy(162)**	**IL-12**	**C8.6**
Dy(163)	CD32abc	FLI8.26
**Dy(164)**	**IFNa**	**LT27/295**
Ho(165)	CD39	eBioA1
Er(166)	CD195 (CCR5)	3A9
Er(167)	CD16	3G8
Er(168)	CD11c	3.9
Tm(169)	CD184 (CXCR4)	12G5
Er(170)	CD14	M5E2
**Yb(171)**	**IL8**	**6265.8**
Yb(172)	CD23	9P25
Yb(173)	CD141	1A4
Yb(174)	CD20	2H7
Lu(175)	CCR7	G043H7
Yb(176)	CADM1	3E 1

Pr: Praseodymium; Nd: Neodymium; Sm: Samarium; Eu: Europium; Gd: Gadolinium; Tb: Terbium; Dy: Dysprosium; Ho: Holmium; Er: Erbium; Tm: Thulium; Yb: Ytterbium; Lu: Lutecium
