# Supplementary material for: Innate cell markers that predict anti-HIV neutralizing antibody titers in vaccinated macaques
Source: Cell Rep Med. 2022 Sep 26;3(10):100751. doi: 10.1016/j.xcrm.2022.100751 (PMC9588994; doi:10.1016/j.xcrm.2022.100751)
Supplement: Document S2. Article plus supplemental information [file mmc2.pdf]

Cell Reports
Medicine

Article

Innate cell markers that predict anti-HIV neutralizing antibody titers in vaccinated macaques

Graphical abstract

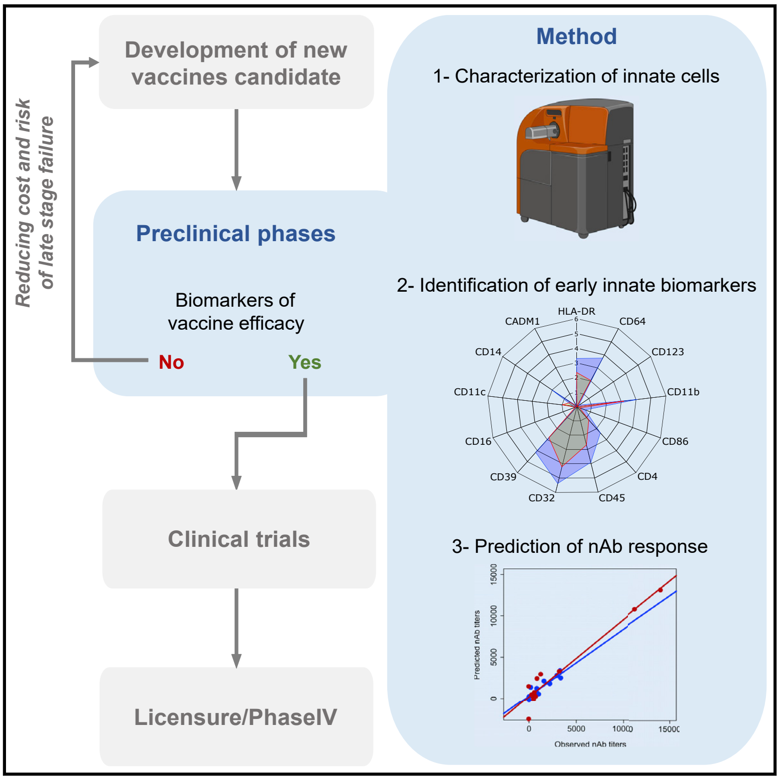

Authors

Matthieu Van Tilbeurgh, Pauline Maisonnasse, Jean-Louis Palgen, ..., Gabriella Scarlatti, Rogier W. Sanders, Roger Le Grand

Correspondence

roger.le-grand@cea.fr

In brief

Van Tilbeurgh et al. use mass cytometry to identify innate cell signature predictive of neutralizing antibody response in a non-human primate model. They highlight that better understanding early immune mechanisms may be of great help to design new vaccine candidates.

Highlights

- HIV-Env SOSIP trimers induce neutralizing antibodies in cynomolgus macaques
- Vaccine-induced innate cells changes are characterized using mass cytometry
- Adjuvant and route of immunization influence early innate signatures in vaccinated NHP
- Early innate cell signatures predict neutralizing antibody levels

Van Tilbeurgh et al., 2022, Cell Reports Medicine *3*, 100751
October 18, 2022 © 2022 The Author(s).
https://doi.org/10.1016/j.xcrm.2022.100751

CellPress

Cell Reports Medicine

CellPress
OPEN ACCESS

Article

Innate cell markers that predict anti-HIV neutralizing antibody titers in vaccinated macaques

Matthieu Van Tilbeurgh,[1] Pauline Maisonnasse,[1] Jean-Louis Palgen,[1] Monica Tolazzi,[2] Yoann Aldon,[3] Nathalie Dereuddre-Bosquet,[1] Mariangela Cavarelli,[1] Anne-Sophie Beignon,[1] Ernesto Marcos-Lopez,[1] Anne-Sophie Gallouet,[1] Emmanuel Gilson,[4] Gabriel Ozorowski,[5] Andrew B. Ward,[5] Ilja Bontjer,[6] Paul F. McKay,[3] Robin J. Shattock,[3] Gabriella Scarlatti,[2] Rogier W. Sanders,[6,7] and Roger Le Grand[1,8,*]

[1]Université Paris-Saclay, Inserm, CEA, Center for Immunology of Viral, Auto-immune, Hematological and Bacterial Diseases (IMVA-HB/IDMIT), 92265 Fontenay-aux-Roses, France

[2]Viral Evolution and Transmission Unit, Division of Immunology, Transplantation and Infectious Diseases, IRCCS Ospedale San Raffaele, 20132 Milan, Italy

[3]Imperial College London, Faculty of Medicine, Department of Infectious Disease, London, UK

[4]Life & Soft, 28 rue de la Redoute, 92260 Fontenay-aux-Roses, France

[5]Department of Integrative Structural and Computational Biology, The Scripps Research Institute, La Jolla, CA 92037, USA

[6]Department of Medical Microbiology and Infection Prevention, Amsterdam University Medical Centers, Location AMC, University of Amsterdam, 1105 AZ Amsterdam, the Netherlands

[7]Department of Microbiology and Immunology, Weill Medical College of Cornell University, New York, NY 10021, USA

[8]Lead contact

*Correspondence: roger.le-grand@cea.fr

https://doi.org/10.1016/j.xcrm.2022.100751

SUMMARY

Given the time and resources invested in clinical trials, innovative prediction methods are needed to decrease late-stage failure in vaccine development. We identify combinations of early innate responses that predict neutralizing antibody (nAb) responses induced in HIV-Env SOSIP immunized cynomolgus macaques using various routes of vaccine injection and adjuvants. We analyze blood myeloid cells before and 24 h after each immunization by mass cytometry using a three-step clustering, and we discriminate unique vaccine signatures based on HLA-DR, CD39, CD86, CD11b, CD45, CD64, CD14, CD32, CD11c, CD123, CD4, CD16, and CADM1 surface expression. Various combinations of these markers characterize cell families positively associated with nAb production, whereas CADM1-expressing cells are negatively associated ($p < 0.05$). Our results demonstrate that monitoring immune signatures during early vaccine development could assist in identifying biomarkers that predict vaccine immunogenicity.

INTRODUCTION

HIV-1 is responsible for a pandemic of more than 37 million people and continues to spread at a rate of >1.7 million new infections every year.[1] It is widely acknowledged that a protective vaccine would be the most effective means to reduce HIV-1 spread and ultimately eliminate the pandemic. Despite decades of research, we do not yet have a vaccine capable of protecting people from HIV-1 infection or halting disease progression. Developing new strategies for HIV vaccines requires a long-term effort challenging the investment in the designing of immunogens and immunization modalities while reducing the risk of failure in the late stages of development.

Vaccine development failure may result from our poor understanding of immune mechanisms of protection following immunization as only a few immune parameters are assessed during efficacy trials. Seeding studies from R.P. Sekali and B. Pulendran have revealed system biology as a promising and powerful exploration tool,[2,3] taking advantage of wide multiplexed data, allowing a deep understanding of cellular and molecular partners implicated in immune responses. Systems vaccinology has been applied to multiple vaccine approaches, including yellow fever, HIV, influenza and recombinant vesicular stomatitis virus–Zaire Ebola virus (rVSV-ZEBOV) vaccines, and to identify adjuvant immune signature.[3–7] Tsang et al. even described host blood parameters, predicting before immunization the capacity to respond to influenza vaccine.[8] Thus, systems vaccinology may contribute to reduce vaccine development failure by identifying markers that predict protective immunity at the early stages of preclinical animal model studies and clinical trials.[9,10]

Studies in animal models are critical in vaccine development. Non-human primates (NHPs) are particularly relevant because of the close phylogenic relationship with humans, resulting in a very similar organization of the respective immune systems, allowing testing immunogenicity without requiring

© 2022 The Author(s).
This is an open access article under the CC BY license (http://creativecommons.org/licenses/by/4.0/).

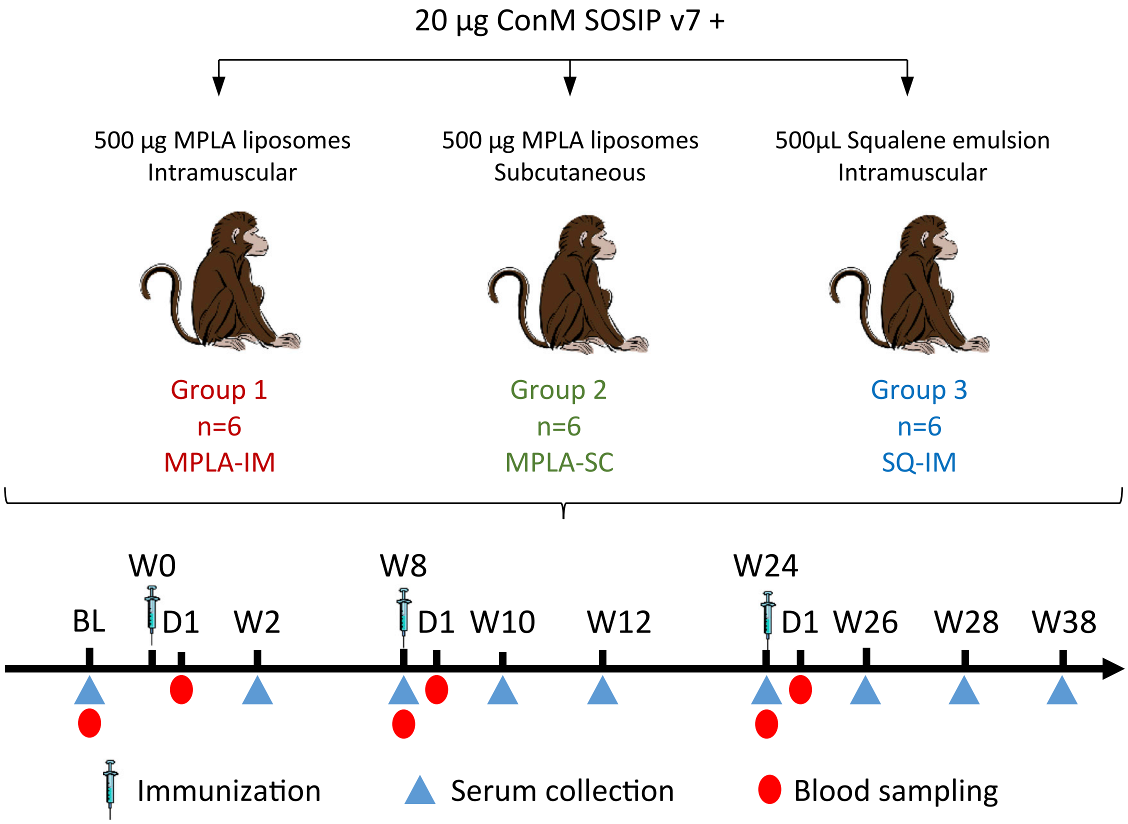

Figure 1. Experimental design

Three groups of six cynomolgus macaques received three immunizations of ConM SOSIP.v7 vaccine adjuvanted either with MPLA liposomes or squalene emulsion and were injected by the intramuscular (IM) or the subcutaneous (SC) route, at week (W)0, W8, and W24. Blood samples were collected before and 24 h after each immunization. Serum was collected before each immunization and then every 2 weeks to quantify Ag-specific IgG, nAb, and FcγR-binding titers.

adaptation of the candidate vaccine to the species. Cynomolgus and rhesus macaques are widely used in the development of an HIV vaccine, because simian immunodeficiency virus (SIV) and simian-human immunodeficiency virus (SHIV) challenge models also recapitulate most of the features of HIV infection and acquired immunodeficiency syndrome (AIDS) in humans.[11,12]

Innate immunity is one of the first players in shaping the vaccine-induced immune responses, and molecular and cellular changes immediately following vaccine injection may help to identify markers predicting the orientation, durability, and efficacy of the adaptive response. Such predictive signals would be particularly useful to accelerate the selection of the most promising vaccine candidates at early developmental stages.

Here, we demonstrate in cynomolgus macaques that subsets of myeloid cells, characterized by mass cytometry and appearing in blood very early following the injection of native-like trimeric HIV-1 envelope SOSIP (Env) immunogens,[13,14] differ depending on the given adjuvant and immunization route. Moreover, we identified a set of cell markers that correlates with vaccine-induced neutralizing antibody (nAb) activity that could be used to develop models that predict the quality of the vaccine response.

RESULTS

The humoral response to an HIV SOSIP Env vaccine is influenced by both the injection route and the adjuvant

We characterized the vaccine-induced response in three groups of six cynomolgus macaques to a stabilized Env derived from a consensus sequence of HIV-1 group M (ConM SOSIP.v7) that we previously reported to induce strong nAb responses in rabbits,[15,16] which is being evaluated in phase 1 trials in humans (NCT03816137, NCT03961438, NCT04046978). We assessed the impact of subcutaneous (SC) or intramuscular (IM) immunization in combination with squalene emulsion (SQ) or monophosphoryl-lipid A liposome (MPLA) adjuvants (Figure 1). The adjuvants were shown not to compromise the conformational integrity of the ConM SOSIP.v7 trimers (Figure S1).

Animals immunized IM with MPLA (Group 1) or SQ (Group 3) showed similar levels of ConM-specific IgG (838,307.004 ± 2.163 and 1,622,973.717 ± 1.737 area under the curve [AUC], respectively [geometric mean ± geometric SD]) and nAb titers (22,610.1922 ± 2.642 and 18,488.409 ± 2.268 AUC, respectively), with peaks at 2 weeks after the first and second boost (week (W) 10 and 26, respectively), suggesting that both adjuvants are as effective by the IM route (Figures 2A–2D and S2). In both groups, ConM-specific IgG levels remained high after

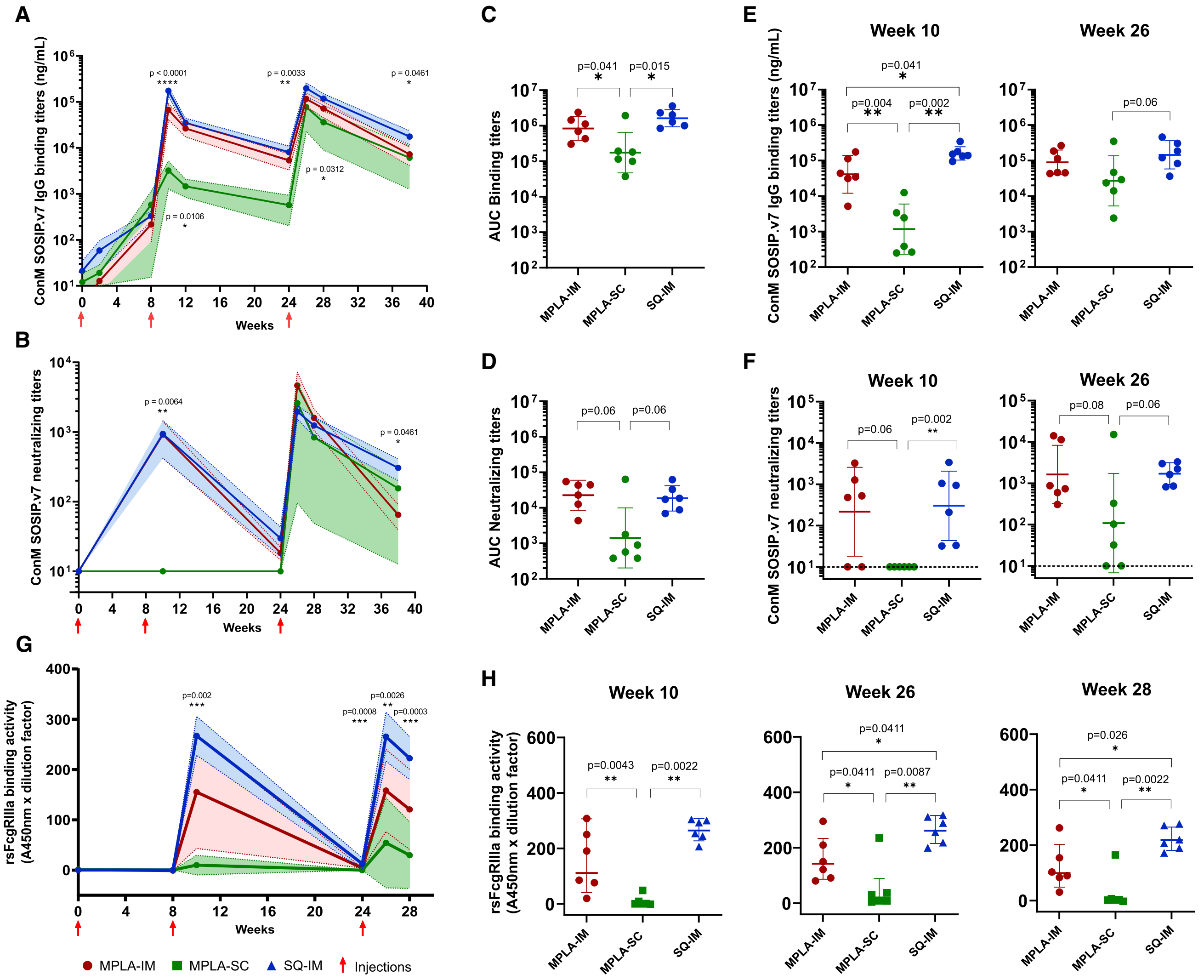

Figure 2. Antigen-specific humoral response in macaques' serum following ConM SOSIP.v7 immunizations

ConM SOSIP.v7 IgG binding (A) and nAb (B) titers. The geometric mean titers and geometric SD of each group are displayed in red (MPLA-IM), green (MPLA-SC), and blue (SQ-IM). Significant differences between groups (n = 6) were assessed with a Kruskal-Wallis test, and p values < 0.05 are displayed.

(C) and (D) display the area under the curve (AUC) from W0 to W38 for ConM SOSIP.v7 IgG binding and nAb titers, respectively. Comparison of ConM SOSIP.v7 IgG binding (E) and nAb titers (F) at the peaks of IgG production (weeks 10 on the left and 26 on the right).

(G) rsFcγRIIIa binding profile of Ag-specific IgG following SOSIP ConM immunizations. The geometric mean titers and geometric SD of each group are displayed.

(H) Comparison of rsFcγRIIIa binding activity geometric mean between vaccines. Pairwise comparison was made using the Mann-Whitney test (n = 6) to compare AUCs, Ab titers, and rsFcgRIIIa binding activity at the different time points. p values < 0.05 are shown. Immunizations (weeks 0, 8, and 24) are indicated by red arrows.

reaching a peak 2 weeks after each boost, while nAb titers dropped after the first (16.288 ± 1.716 and 20.246 ± 2.461 ng/mL, respectively) and to a lesser extent after the second boost (41.296 ± 2.906 and 202.250 ± 2.945 ng/mL, respectively). In contrast, MPLA-SC immunization (Group 2) induced significantly lower ConM-specific IgG levels (1421.370 ± 7.028 AUC) compared with MPLA-IM and SQ-IM immunizations (p = 0.041 and p = 0.015, respectively, Figure 2C), especially at W10 (Figure 2E). Neutralization to conM was observed in the MPLA-SC group only after the third immunization with heterogeneous nAb titers. Notwithstanding, neutralizing titers tended to be lower in the MPLA-SC group despite two animals displaying equivalent titers to the lowest and highest of the IM groups (Figure 2F). Heterologous neutralization against the highly neutralization-sensitive 93MW965.26 (MW965) pseudotyped virus (PSV) at W28 was also assessed. Neutralization of MW965 was 10- to 100-fold lower than that of ConM, but similar trends were observed between groups (Figure S3).

We then compared Fcγ-receptor IIIa (FcγRIIIa) binding characteristics of ConM SOSIP.v7-induced serum Ab between the three groups. This has been shown to provide an indirect readout for Ag-specific antibody-dependent cellular cytotoxicity

(ADCC).[17] Similarly to nAb titers, a sharp increase of recombinant soluble (rs) FcγRIIIa binding activity was observed in both IM groups after each boost followed by a decrease. We observed that the SQ formulation generated higher rsFcγRIIIa binding levels than MPLA by the IM route with both groups inducing significantly higher rsFcγRIIIa binding activity than the MPLA-SC induced (Figure 2G). Although the SQ-IM group displayed a more homogeneous response, levels of binding became significantly higher than the MPLA-IM group after the third injection (W26, p = 0.0411; W28, p = 0.026) (Figure 2H). These results highlight that not only the route but also the adjuvant impact rsFcgRIIIa binding activity. Overall, we demonstrated that ConM SOSIP.v7-specific humoral responses were more efficiently induced by the IM than the SC route.

Both the adjuvant and route of immunization affect myeloid cell dynamics

The route- and adjuvant-dependent differences we observed in the anti-Env antibody response argue for a role of early innate immunity in shaping the nAb response. Here, we focused on the myeloid cell compartment because of its role in capturing and presenting vaccine antigens to B and T lymphocytes, in addition to its contribution to inflammation. Mass cytometry was used to extensively characterize the differentiation and functional markers in the heterogeneous blood cell population. Importantly, many of the cell markers we studied in cynomolgus macaques have human counterparts, emphasizing the relevance of this species as a model for evaluating human candidate vaccines. In addition, the antibody panel used in our study consisted of anti-human antibodies cross-reacting with macaque cell markers, thus facilitating the translatability of preclinical study protocols to human clinical trials samples.

A spanning-tree progression analysis of density-normalized events (SPADE) was performed on mass cytometry-generated data to identify leukocyte clusters displaying similar phenotypes (Figure S4A). Major cell populations were defined based on CD3, CD4, CD8, CD20, and HLA-DR expression, which allowed for the identification of 225 myeloid cell clusters (Lin$^-$ HLA-DR$^+$). Based on cell population dynamics following vaccine injection, MPLA-IM induced stronger mobilization of total myeloid cells than the other adjuvants and routes within the first 24 h (Figures S4B–S4D). Granulocytes were not evaluated in this study due to the technical limitation of using frozen and thawed whole blood samples. To better visualize the cluster phenotypes, we then performed hierarchical clustering based on their relative marker levels (Figure 3). Clusters displaying similar phenotypes were gathered into 28 phenotypic families (PFs), described in Table 1. The cluster dendrogram divides the PFs into three superfamilies: (1) HLA-DR$^+$ CD14$^+$ CD11b^{hi}, which we identified as monocytes, (2) HLA-DR$^{mid/hi}$-CD14$^-$-CD11c$^-$, which we identified as a subset of dendritic cells (DCs), and (3) HLA-DR$^{lo/mid}$-CD14$^-$-CD11c$^+$-CD16$^+$, which we identified as another subset of DCs. We determined the impact of ConM SOSIP.v7 immunization on these populations by investigating the differences in cell abundance at different time points after vaccination (Figure 4A and S5 and Table S1). The MPLA-IM group showed significant changes in the myeloid compartment with the frequency of nine PFs significantly increased as early as 24 h following the first injection (Figure 4A and Table S1). These changes affected monocyte populations with profiles of classical monocytes (PF10, p = 0.03), non-classical monocytes (PF19, p = 0.03), intermediate monocytes (PF22, p = 0.03), and moDCs (PF2, PF15, PF25, p = 0.03). These last PFs displayed the most significant changes through time for all vaccines, especially for MPLA-IM (Figure 4B). The first injection also induced an increase in the frequency of cDC1-like cells (PF28, p = 0.03; PF16, p = 0.03) and HLA-DR^{lo}-CD11c^{hi}-CD16^{hi} DCs (PF14, p = 0.03). The first boost injection only mobilized monocyte populations (PF10, PF15, PF2, and PF25, p = 0.03). Finally, the second boost mobilized a much wider range of myeloid cells. In addition to the monocyte families, there was an increase in the frequency of cells with a macrophage phenotype (PF27, p = 0.03), cDC1-like cells (PF28, PF26, and PF16, p = 0.03), and HLA-DR^{lo}-CD11c^{hi}-CD16^{hi} DCs (PF17, PF4, PF5, PF14, PF6, PF18, and PF7, p = 0.03). Overall, the use of MPLA by the IM route appeared to mainly recruit and elicit monocyte/macrophage populations, with an important extension to DC populations after the third vaccine injection, compared with previous time points, indicating that there are host changes over time that affect the quality of the innate response.

The MPLA-SC group mostly displayed changes within the monocyte compartment. MoDCs (PF2, PF15, and PF25, p = 0.03) were the only subsets significantly induced 24 h after the first injection, along with a cDC2 subpopulation (PF24, p = 0.03). Similar to MPLA-IM, the second immunization mobilized classical monocytes (PF10, p = 0.03) and moDCs (PF2, PF15, and PF25, p = 0.03) as well as intermediate monocytes. Finally, seven PFs showed significant changes after the last immunization, with increasing frequencies of monocyte populations (PF2, PF15, PF22, PF25, p = 0.03), as well as cDC2 (PF24, p = 0.03) and HLA-DR^{lo}-CD11c^{hi}-CD16^{hi} DCs (PF4, p = 0.03). Conversely, the PF23 pDC subset showed a significant decrease relative to the baseline level. Thus, SC injection of MPLA shows similar monocyte mobilization as MPLA-IM but of lower amplitude. We found no significant amplification of DCs in the MPLA-SC group, highlighting the influence of the administration route on the quality of the innate response, despite the use of the same adjuvant.

Few significant differences were observed for this group when compared with the SQ-IM group. Most changes after the first injection occurred within the monocyte compartment (PF1, PF2, PF3, and PF19) except for the PF11 HLA-DR$^{mid/hi}$-CD14$^-$-CD11c$^-$ DC subpopulation. The frequency of classical PF1 and PF3 monocytes, non-classical PF19 monocytes, and PF11 cDC1 decreased after ConM SOSIP.v7 injection, whereas that of PF2 moDCs showed a marked increase relative to baseline levels. The second injection only mobilized PF2 moDCs, and we observed a loss of PF11 cDC1 and PF16/PF17 HLA-DR^{lo}-CD11c^{hi}-CD16^{hi} DCs. Finally, similar to groups 1 and 2, the final injection induced strong mobilization of PF2, PF15, and PF25 moDCs.

Vaccine-induced blood cell signatures

We identified vaccine signatures by performing linear discriminant analysis (LDA) (Figures 5A and 5B). We grouped PFs sharing close profiles of changes over time into eight kinetic families

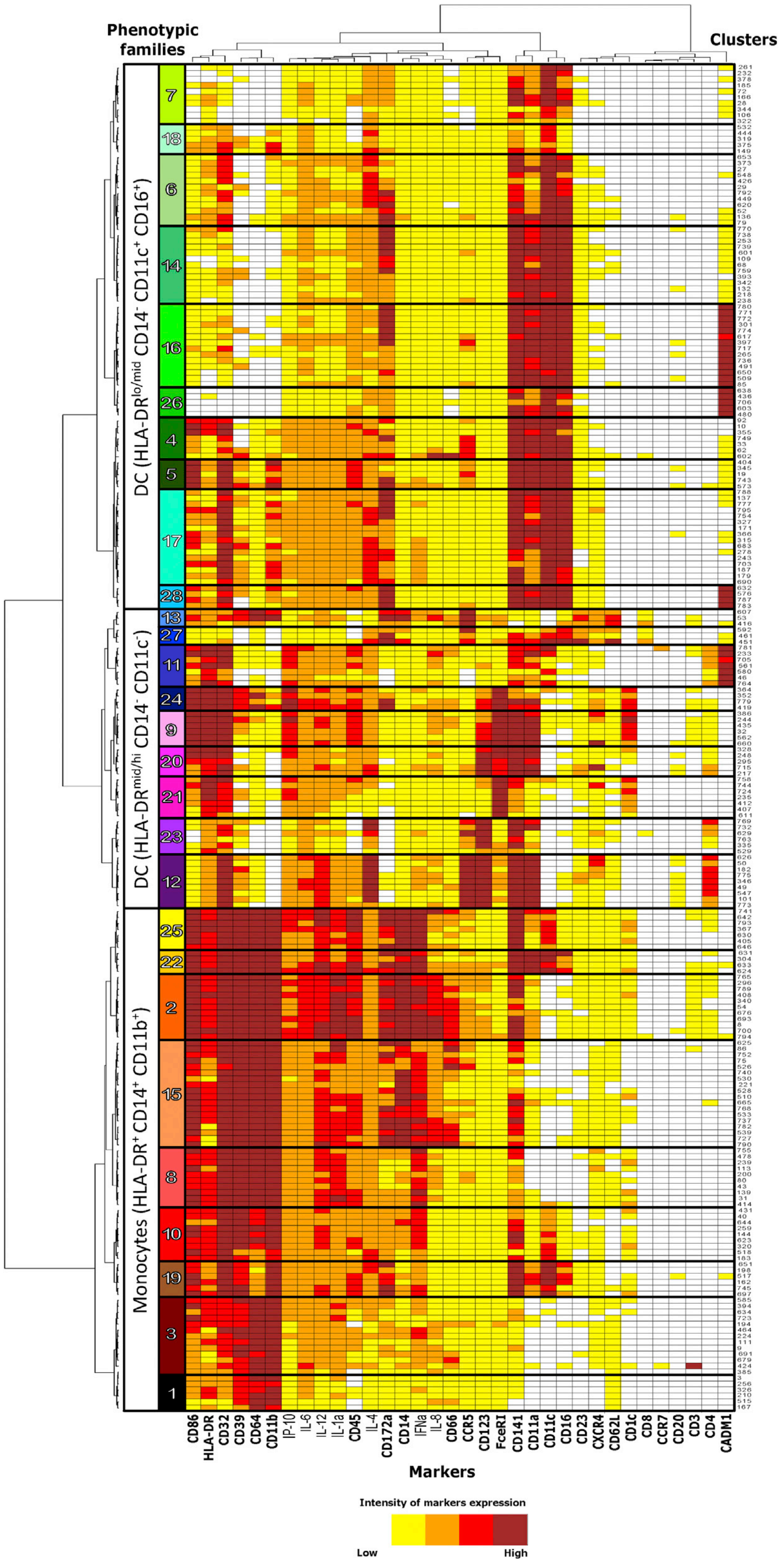

Figure 3. Phenotypic diversity of the blood myeloid cell compartment

The heatmap shows the hierarchical clustering and gathering of all myeloid cell SPADE clusters. Marker expression is shown in columns and cell clusters in rows. Twenty-eight phenotypic families were defined by proximity in the cluster dendrogram and manually annotated. The marker dendrogram represents markers with similar expression patterns. Markers used by SPADE unsupervised analysis for the clustering are written in bold.

Table 1. Characterization of the main myeloid cell populations

Kinetic families	Population characteristics	Phenotypic families	Subsets characteristics	Cell population*	References
KF-I	HLA-DR^{+}, CD64^{hi}, CD86^{hi}, CD11b^{hi}	1	CD14^{lo}, CD32^{mid}	classical monocytes	Nakaya et al. [7]; Tsang et al. [8]; Trautmann and Sekaly[9]; Mooney et al.[10]; Reimann et al.[11]; Estes et al.[12]; Sanders and Moore[13]; De Taeye et al.[14]; Sliepen et al.[15]; Brouwer et al.[16]
		3	CD14^{lo}, CD32^{+}		
		10	CD14^{lo}, CD32^{hi}		
		15	CD14^{+}, CD1c^{lo}	MoDC	
		25	CD14^{+}, CD1c^{lo}		
		19	CD14^{lo}, CD16^{+}, CD11c^{+}	non-classical monocytes	
		22	CD14^{+}, CD16^{+}, CD11c^{+}	intermediate monocytes	
KF-II	HLA-DR^{hi}, CD32^{hi}, CD64^{hi}, CD86^{hi}, CD11b^{hi}	2	CD14^{+}, CD1c^{lo}	MoDC	Reimann et al.[11]; Zhang et al.[18]
	HLA-DR^{+}	20	FceRI^{hi}, CD32^{hi}, CD86^{hi}, CD4^{lo}	putative cDC2	Reimann et al.[11]; De Taeye et al.[14]; Wines et al.[17]; Calabro et al.[19]
		9	CD1c^{+}, FceRI^{hi}, CD32^{hi}, CD86^{hi}, CD4^{lo}	cDC2	
		24			
	HLA-DR^{mid}, CD11c^{+}, CD16^{+}, CD32^{+}	28	CADM1^{+}	cDC1 like DC	De Taeye et al. [14]; Wines et al.[17]
KF-III	HLA-DR^{var}, CD11c^{+}, CD16^{+}, CD32^{+}	4	/	DC	/
		5			
	HLA-DR^{lo}, CD11c^{hi}, CD16^{hi}	6	/	DC	/
		7			
		14			
	HLA-DR^{lo}, CD11c^{hi}, CD16^{lo}	18	CD11a^{lo}		
KF-IV	HLA-DR^{+}, CD32^{hi}, CD64^{hi}, CD86^{hi}, CD11b^{hi}	8	CD14^{lo}	classical monocytes	Nakaya et al.[7]; Tsang et al.[8]; Trautmann and Sekaly[9]; Mooney et al.[10]; Reimann et al.[11]; Estes et al.[12]; Sanders and Moore[13]; De Taeye et al.[14]; Sliepen et al.[15]; Brouwer et al.[16]
	HLA-DR^{var}, CD11c^{+}, CD16^{+}, CD32^{+}	17	/	DC	/
KF-V	HLA-DR^{+}	11	CADM1^{+}, CD1c^{-}, CD172a^{lo}, CD16^{lo}	cDC1	De Taeye et al.[14]; Wines et al.[17]

(*Continued on next page*)

Table 1. *Continued*

Kinetic families	Population characteristics	Phenotypic families	Subsets characteristics	Cell population*	References
		21	$CD1c^{+}$, $FceRI^{hi}$, $CD86^{mid}$ $CD123^{-}$, $CD4^{-}$	immature cDC2	Pauthner et al.[20]
KF-VI	$HLA\text{-}DR^{lo}$, $CD123^{+}$	12	$CD11a^{hi}$, $CD4^{+}$, $CCR5^{+}$	pDC	Reimann et al.[11]; Estes et al.[12]; Wines et al.[17]; Pauthner et al.[20]; Calabro et al.[19]; O'Hagan et al.[21]; Rosenbaum et al.[22]; Anderson et al.[23]; Sanders et al.[24]
		23	$CD11a^{var}$, $CD4^{lo}$, $CCR5^{var}$	pDC/blood DC precursor	
KF-VII	$HLA\text{-}DR^{lo}$, $CD14^{+}$, $CD11b^{+}$, $CD64^{+}$, $CD11c^{-}$, $CD16^{-}$	13	$CCR5^{+}$, $CXCR4^{+}$, $CD62L^{+}$	putative macrophages	/
	$HLA\text{-}DR^{mid}$, $CD14^{+}$, $CD11b^{-}$, $CD64^{-}$, $CD11c^{+}$, $CD16^{+}$	27	$CCR5^{+}$, $CXCR4^{+}$, $CD62L^{+}$	putative macrophages	/
KF-VIII	$HLA\text{-}DR^{lo}$, $CD11c^{hi}$, $CD16^{hi}$	16	$CADM1^{+}$	cDC1 like DC	De Taeye et al.[14]; Wines et al.[17]
		26			

The phenotypic composition of each kinetic family is detailed. The main phenotypic characteristics used to identify cell populations according to the expression profile shown in the heatmap (Figure 3) are indicated. *proposal of cell annotation. Absence of data were indicated with /.

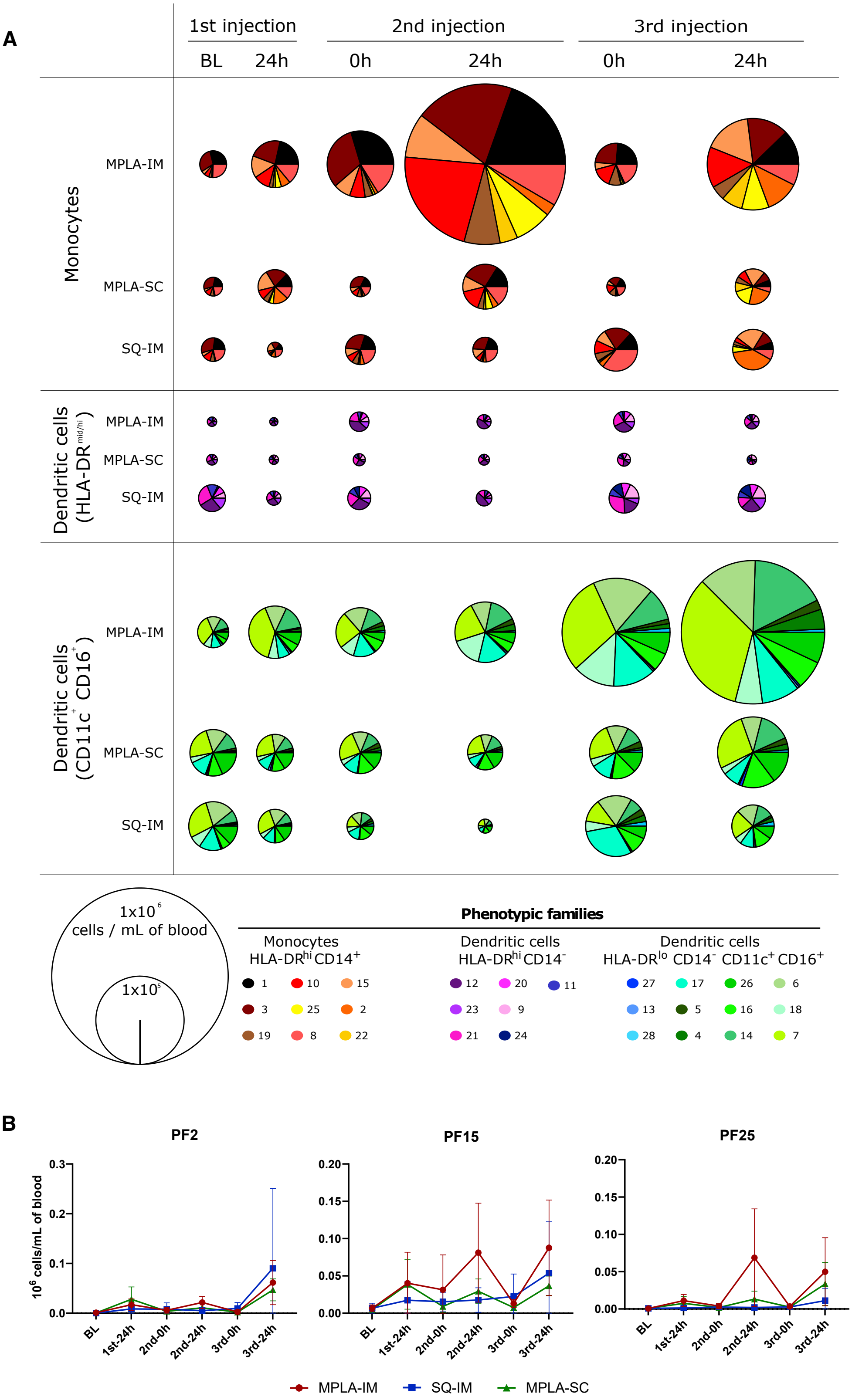

(legend on next page)

(KFs, Table 1), taking into account the correlation between variations in abundance among PFs (Figures S6 and S7 and Table S2). LDA differentiated the vaccine regimens for every time point (Figure 5A). Then, a second LDA was performed to identify KF associated to the vaccine regimens (Figure 5B). In this model, KF-III (CD11c$^+$-CD16$^+$ DCs) was associated with MPLA-IM. Similarly, KF-VIII was associated with MPLA-SC, composed of two PFs (16 and 26) belonging to CD11c$^+$-CD16$^+$ DCs and showing high expression of CADM1, suggesting a cDC1-like signature. By contrast, SQ-IM was characterized by highly activated moDCs (PF2) and cDC2 (PF9, 20, 21, and 24) from KF-II but also immature cDC2 from KF-V and pDCs from KF-VI. Additionally, the LDA score of KF-I monocytes (PF1, 3, 10, 15, 19, 22, and 25), KF-VII (PF13 and 27), and KF-IV (PF8 and 17) did discriminate SQ-IM from MPLA vaccines but could not be associated either with MPLA-IM or MPLA-SC, suggesting an MPLA adjuvant signature for those KFs.

Next, we identified the main phenotypic differences between cell populations using the Kolmogorov-Smirnov distance (Figure 5C). The expression of HLA-DR, CD11b, CD45, CD64, CD39, CD14, CD86, and CD32, indicative of a highly activated state, was significantly stronger for monocytes belonging to the SQ-IM signature than to the MPLA signature. MPLA injected via the IM route induced DCs expressing lower levels of HLA-DR, CD39, CD123, and CD86, but higher levels of CD11c and CD16 than SQ injected via the same route (SQ-IM group). Finally, the SC route (MPLA-SC group) appears to differ from the IM route through high CADM1 expression.

In conclusion, each vaccine strategy induced a unique innate cell signature (Figure 5D), allowing to discriminate between vaccine routes and adjuvants and defined by a combination of a small set of markers: HLA-DR, CD39, CD86, CD11b, CD45, CD64, CD14, CD32, CD11c, CD4, CD16, CD123, and CADM1.

Association of innate and adaptive immunity

We then investigated whether this set of markers could be used to predict the magnitude of the nAb response, IgG binding response, and Fc-mediated effector functions of IgGs (Figure 2). A generalized linear model constructed with KF abundances and antibody titers, neutralizing or FcγRIIIa functions, measured 2 weeks after the second and third injection, was employed as predictive model (Figure 6).

Interestingly, three KFs for MPLA-IM (KF-II, III, and VII) and four for SQ-IM (KF-III, V, VII, and VIII) significantly correlated with nAb production ($p < 0.05$). KF-II, characterized by high expression of moDC and cDC2 markers (Figure 5D), was the family that most positively associated with high nAb titers for the MPLA-IM (Figure 6A). These cells are reported to display strong CD4$^+$ T cell priming activity, notably for T helper 2 (Th2) cells, known to be involved in Ab responses. Consistently, this family was also positively associated with IgG binding titers for MPLA-SC and was not associated with IgG binding titers for MPLA-IM (Figure 6C). KF-III composed of HLA-DR^{lo}-CD11c$^+$-CD16$^+$ DC was negatively associated to nAb production (p = 0.004) but was positively associated with rsFcγRIIIa binding activity along with KF-V (Figure 6E).

For the SQ-IM group, and in marked contrast to MPLA-IM, the model positively associated KF-III with the nAb titer (Figure 6A) and monocytes from KF-I with rsFcγRIIIa binding activity (Figure 6E). KF-VII, presumably composed of macrophage populations based on secondary markers expression, was also positively associated with nAb production, IgG, and rsFcγRIIIa binding activities (Figures 6A, 6C, and 6E, respectively) for the SQ-IM group. Two KFs were negatively associated with SQ-IM nAb titers, both sharing similar features. Indeed, KF-V and KF-VIII both showed high CADM1 expression, mostly attributed to the cDC1 phenotype and cytotoxic response (Figure 6A). The CADM1$^+$ cells of KF-V were HLA-DR^{hi}-CD11c$^-$-CD16$^-$, implying a cDC1 phenotype, whereas cells belonging to KF-VIII showed the opposite pattern of HLA-DR^{lo}-CD11c$^+$-CD16$^+$ expression, characteristic of cDC1-like cells (Figure 5D). CADM1$^+$ cells from KF-VIII were also negatively associated with IgG binding for both MPLA-SC and SQ-IM group and with rsFcγRIIIa for all three groups (Figures 6C and 6E).

The robustness and accuracy of the model were confirmed by correlating predicted and observed values for every immune parameter studied (Figures 6B, 6D, and 6F). The observed nAb titers strongly correlated with those predicted by the model for both the MPLA-IM and SQ-IM vaccine strategies ($r = 0.97$ and $r = 0.9$, respectively). A strong correlation of IgG binding titers was also found for SQ-IM and MPLA-SC ($r = 0.89$ and $r = 0.92$, respectively) (Figure 6D) as well as MPLA-IM and SQ-IM with rsFcγRIIIa binding activity ($r = 0.9$ and $r = 0.75$, respectively) (Figure 6F). However, no reliable prediction could be made regarding MPLA-SC nAb titers and rsFcγRIIIa binding activity because they were only observed at low level after the third injection and not in all individuals.

These results demonstrate the ability of our model to predict humoral response, including IgG binding titers, nAb activity, and FcγRIIIa engagement, through the characterization of innate cell populations as early as 1 day after vaccine injections.

DISCUSSION

Vaccine parameters, such as adjuvant and administration route, strongly influence the quality of the innate immune response and, ultimately, the adaptive immune response.[18–22] NHPs are particularly relevant for such translational immunology studies because of the high similarity between the organization of the macaque and human immune systems. Here, we demonstrate in NHPs that it is possible to anticipate the quality of specific antibody responses based solely on the early innate response. We show that autologous ConM SOSIP.v7-specific IgG binding titers, nAb

Figure 4. Follow-up of monocytes and dendritic cells population enrichment

The pie charts show the average phenotypic family cell abundance between immunization groups within the monocyte and dendritic cell compartments (A). Phenotypic families are represented using the same colors and numbers as in Figure 3. The cell numbers correlate with the size of the segments in the pie charts and are given as 10^6 cells per mL of blood.

(B) Mean cell abundances dynamic and SD from the top three phenotypic families that display the most significant variations over the different time points. p values for the changes are presented in Table S1 and the fold change in Figure S5.

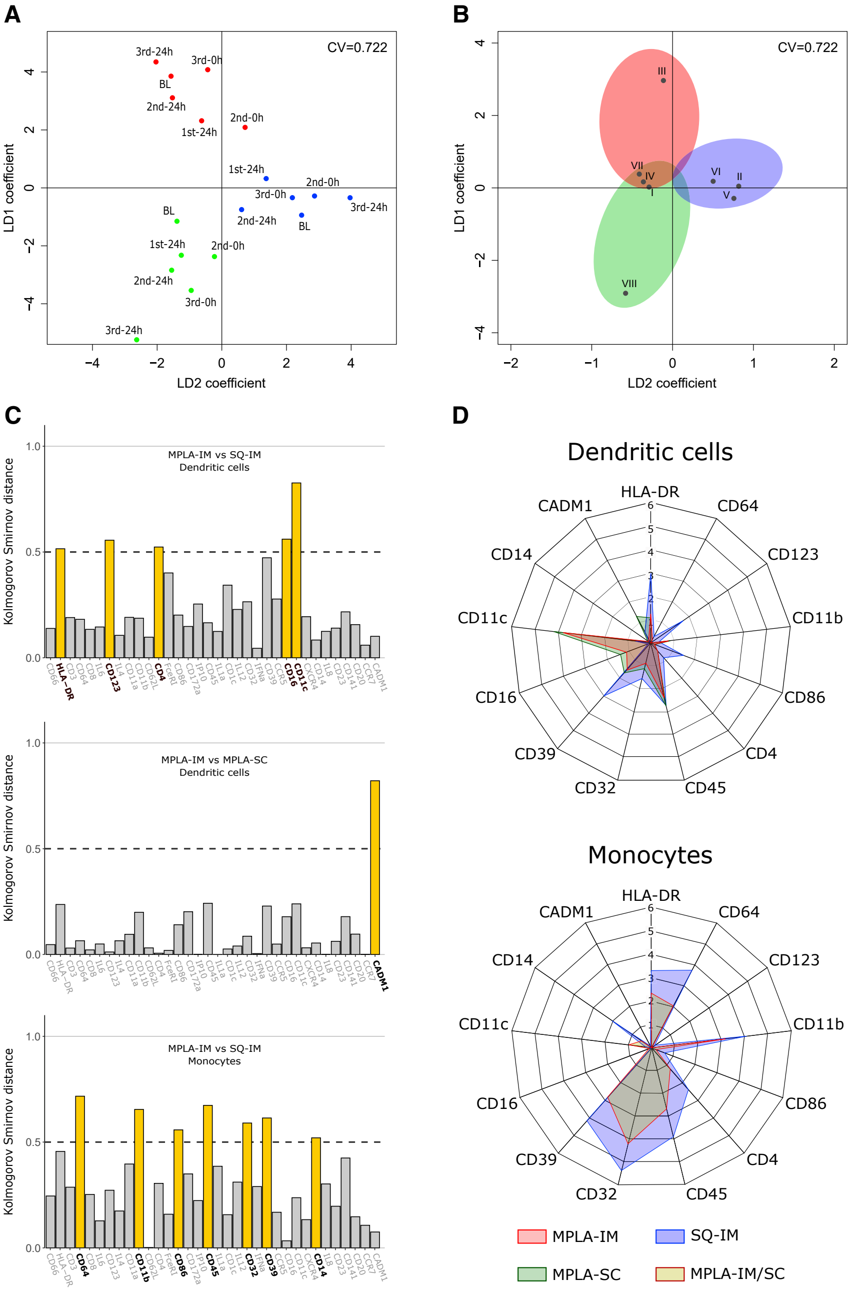

Figure 5. Identification of vaccine cell signatures

(A) Linear discriminant analysis in two dimensions (LD 1 and LD 2) performed using mean kinetic family abundances of all individuals to discriminate vaccine regimens at each time point.

(B) LDA analysis conducted with the mean abundances of KF abundances for all individuals and all time point to identify the contribution of each KF to discriminate vaccine regimens. The colored areas show the association of kinetic families with the MPLA-IM (red), MPLA-SC (green), and SQ-IM (blue) injections. The areas are positioned according to the LDA results in (A) separating the different vaccine regimens. Kinetic families for which the Euclidian distance to the origin did not reach the threshold of 0.5 (black circle) were not considered as discriminant between vaccines.

(C) Identification of the vaccine-associated KF (defined in B) markers that contribute the most to discriminate vaccine regimens. The differential expression for each marker between vaccines is quantified by the Kolmogorov-Smirnov distance indicating the maximal distance between cumulative distribution function (CDF). DCs and monocytes from each signature were separated to establish distances and the threshold used to identify discriminant markers were represented with dot lines.

(D) Median signal intensity (MSI) distributions of markers previously revealed in (C) for each population belonging to vaccine signatures, with MPLA-IM in red, MPLA-SC in green, and SQ-IM in blue. Monocyte signatures for both MPLA injections overlap, as they are characterized by the same kinetic families.

production, and rsFcγRIIIa binding activity are induced more efficiently by the IM than the SC route when considering MPLA. However, the nAb responses induced by ConM SOSIP.v7 decayed quickly, which is consistent with many previous observations using HIV-1 Env-based vaccines, including gp120s and SOSIP trimers.[23,24] These observations imply that HIV-1 Env-based vaccines are poorly able at generating long-lived plasma cells and should guide strategies to improve the durability of anti-Env immunity. We have previously studied the Ab specificities induced by the ConM SOSIP.v7 immunogen in rabbits and determined that the dominant nAb response is directed against a region involving V1, V2, and V3.[15,25]

Discrepancies between adjuvants occurred especially after the third injection, where SQ-IM vaccinated animals displayed a higher and more homogeneous rsFcγRIIIa binding activity than MPLA-IM, suggesting that SQ-IM ConM SOSIP.v7 vaccines have better abilities to induce FcR-mediated effector functions. Though, a SQ-SC group would be of interest to verify if IM induced more efficient humoral responses than SC independently of the adjuvants and to confirm our hypothesis.

There are reported evidences that early events following vaccine injections affecting APC targeting, activation, and antigen processing and presentation impact the quality of adaptive response. Indeed, Schifanella et al. reported in the NHP model that "The relationship between vaccine efficacy and the neutralization profile of the challenge virus appear to be linked to the different immunological spaces created by MF59 and Alum via CXCL10 and IL-1β, respectively,"[26] suggesting a role for innate cells in the quality of induced nAb. This study confirms observations of the same group of authors who demonstrated that "Vaccine efficacy was associated with alum-induced, but not with MF59-induced, envelope (Env)-dependent mucosal innate

CellPress
OPEN ACCESS

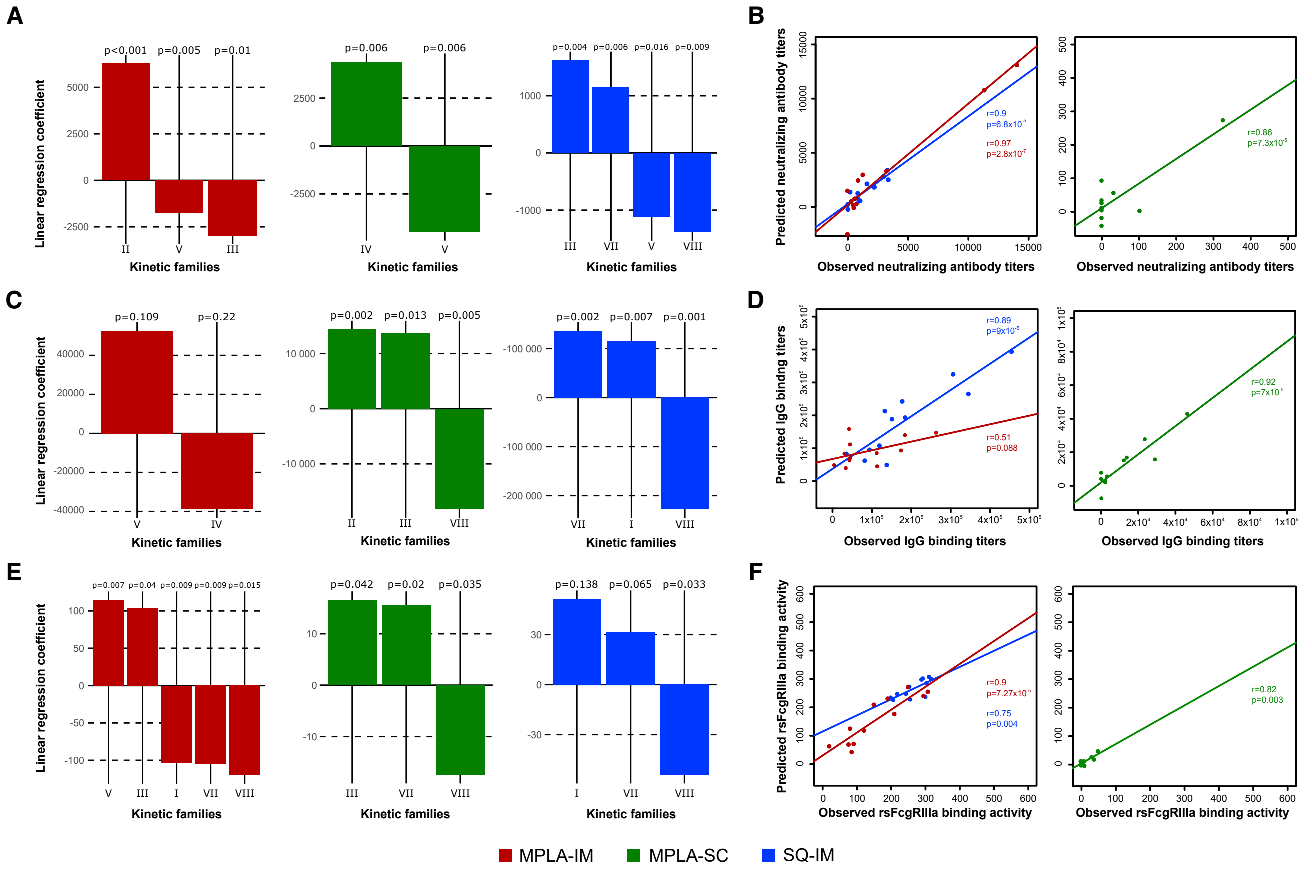

Figure 6. The abundance of innate immune cell populations predicts ConM SOSIP.v7 humoral responses

A linear regression was employed to assess the relationship of the kinetic family cell abundance 24 h after the second and third injections with three biological outcomes: the nAb titer (A), the IgG binding titer (C), and the rsFcγRIIIa binding activity (E). MPLA-IM, MPLA-SC, and SQ-IM are displayed in red, green, and blue respectively. Linear regression coefficients with a p value $\leq$ 0.05 are considered necessary and sufficient to predict those humoral parameters. Iterative linear regressions were generated until all coefficients had a p value $\leq$ 0.05. (B, D, and F) represent the correlation of the observed and predicted values by the linear regression for nAb titer, IgG binding titer and the rsFcγRIIIa binding activity, respectively. The Pearson correlation coefficient and p values are shown.

lymphoid cells (ILCs) that produce interleukin (IL)-17, as well as with mucosal IgG to the gp120 variable region 2 (V2) and the expression of 12 genes, ten of which are part of the RAS pathway."[27] Similarly, the route of administration, and therefore the type of locally targeted APC, has been described as an important factor driving the quality of the neutralizing response against an SOSIP protein. Pauthner et al. demonstrated that subcutaneous immunizations induce stronger nAb responses against tier-2 HIV than intramuscular immunizations. Contrary to this study, we demonstrated that intramuscular immunizations elicit higher nAb responses against the SOSIP protein. The use of ISCOMATRIX as adjuvant by Pauthner et al. may lead to a different APC targeting, activation, and antigen processing and presentation then generating different nAb responses from what we observed.

Our results suggest that MPLA mainly affects the expansion of monocyte, moDC, and HLA-DR^{lo}-CD11c^{+}-CD16^{+} DC populations, especially when combined with the IM route. This may reflect adjuvants' properties to mobilize immune cells into tissues as shown in mice with MF59, an oil in water emulsion similar to SQ.[28] Nevertheless, adjuvants alone could not explain the differences we observed. Indeed, the quality of innate immune responses is also dependent on immune cells tissues colonization, as previously reported.[22] Ols et al.[29] showed that an IM injection induced a greater antigen uptake by antigen-presenting cells than an SC injection of Env proteins in MPLA liposomes. They hypothesized that this may be due to a better formation of immune complexes when subjects are re-exposed because of the high vascularization of muscles. Such an effect, combined with the increasing number of circulating moDCs and HLA-DR^{lo}-CD11c^{+}-CD16^{+} DCs following ConM SOSIP.v7 immunization, could explain the higher nAb titers we observed following IM injections. Strikingly, the later monocyte activation and higher proportion of CADM1^{+} cells observed for the MPLA-SC compared with the other vaccine strategies may also be involved in the delayed nAb production observed in this group.

The dynamic changes observed for HLA-DR^{lo}-CD11c^{+}-CD16^{+} DCs following the MPLA-IM injections may also be associated with the properties of the tissue, as we previously showed a higher proportion of CD11c^{+} DC infiltration in the muscle than

in the skin following an SC modified vaccinia Ankara (MVA) vaccine injection.[22]

The panel we used lacked antigen targeting receptors to address the antigen presentation capacities of DC, but the vaccine-induced signatures on all other markers and prediction analysis we performed may indeed help to speculate on DCs functionalities. In our study, cDC2 was identified as the SQ-IM vaccine group signature and was also positively associated with nAb production for MPLA-IM vaccine. However, the low abundance of variations detected suggests that cDC2 alone may not be the only APC implicated in the functional outcomes. The PF15 and PF25, identified as moDCs— known to have potent antigen presentation capacities—were also strongly mobilized by MPLA-IM and SQ-IM vaccines. Highly activated moDCs from PF2 were also recruited by the two groups and the SQ-IM group in particular. In addition to its similarity of phenotypic profile with cDC2, moDC expresses DC-SIGN and the macrophage mannose receptor, two receptors implicated with antigen sensing and induction of Th2 responses and then able to drive immune response toward antibodies production.[30]

Noteworthy, studies on antigen delivery in mice demonstrated that targeting CD11c led to the induction of strong $CD4^+$ and $CD8^+$ T cells response in addition to strong antibody responses.[31,32]The $CD11c^+$ DC subsets abundance we observed suggest its key role in vaccine response orientation. This may be particularly true for MPLA-IM group where some of $CD11c^+$ DC kinetics families (KF-III) constitute the vaccine signature.

On the other hand, the MPLA-SC group can be discriminated from the two other route/adjuvant combinations based on CADM1 expression by cDC1, which is classically associated with $CD8^+$ T cells and Th1 priming. In addition, cDC1 subsets we identified also express Clec9A, a C-type lectin receptor known for its implication in the generation of cytotoxic CD8 T cell responses. We may thus speculate that if not optimal for antibody production, this adjuvant/route combination would be of interest for promoting cellular immunity. The key role of CADM1 in vaccine response orientation is conferment by the negative association we observed for this marker with MPLA-IM and SQ-IM in induced antibody responses.

FcγRIIIa and FcγRIIIb, two isoforms of CD16, have been associated with innate cell cytotoxicity.[33] Monocytes are known to express CD16, and although $CD16^+$ DCs remain a controversial subset, Fromm et al.[34] demonstrated that $CD16^+$ monocytes and $CD16^+$ DCs were two phenotypically and functionally distinguishable populations that have been identified to be able to perform ADCC.[35,36] Our data show an important mobilization of monocytes and $CD16^+$ DCs in the blood of all animals, and especially for the MPLA-IM group. However, a greater rsFcγRIIIa binding activity was observed in the SQ-IM vaccinated animals, where those populations were less represented. This suggests that rsFcγRIIIa binding activity may not be exclusively explained by monocytes and $CD16^+$ DCs recruitment. Indeed, other $CD16^+$ innate populations, such as neutrophils and NK cells, known to exert cytotoxic activity, might have been underestimated in our study.[37] We recently reported that polymorphonuclear (PMN) cells, in particular, are key players in vaccine responses.[38,39] Unfortunately, granulocytes were unexplored in our study because of technical limitations. Future studies should assess the value of integrating PMN and NK markers in such kind of analysis, in order to correlate these populations to rsFcγRIIIa binding activity and thus more finely predict vaccine responses.

In addition to PMN and NK cells, using similar approaches for the study of B and T lymphocytes, including antigen-specific cells, would be of interest to characterize the installation of vaccine response, as it has been shown that increased antibody responses depend on enhanced interactions between APCs and $CD4^+$ T cells and B cells.

In the field of HIV vaccines, an important role of myeloid cell-expressing markers was also identified in our NHP study. Indeed, in a human systems vaccinology approach of the ALVAC-HIV vaccine, Andersen-Nissen and colleague observed an enrichment of monocytes and $CD16^+$ monocytes-associated transcripts.[40] Also, NHP studies based on the ALVAC-SIV have associated monocytes signatures with a decreased risk of SIVmac251 acquisition.[27,41] In our manuscript, we also highlighted an important role of monocytes following the different vaccines including $CD16^+$ non-classical and intermediate monocytes, especially for animals immunized with the HIV glycoprotein in MPLA adjuvant and by IM route. Furthermore, we also evidenced a role for $CD11c^+$-$CD16^+$ DCs that was not mentioned in these previous studies.

It is possible that the changes in cell populations we observed in the cynomolgus macaque model may be of interest in the design and monitoring of vaccine responses in humans, although species specificities in immune cell compartments need to be considered when transferring knowledge from animal studies to clinical trials.[42–44]

In conclusion, we show that autologous Ab binding titers and neutralizing activity are induced more efficiently by the IM than SC route when MPLA is used as adjuvant and that the efficacy of MPLA and SQ is in the same range when the IM route is used, even though immune pathways involved seem to differ. We also reveal an association between early-occurring myeloid cell signatures following immunization and adaptive immune parameters. These findings provide new insights on immune mechanisms of interest for vaccine innovation and demonstrate that monitoring of innate signatures during early vaccine development could help minimizing the risk of failure during clinical development phases.

Limitations of the study

The results from this study may inform future strategies for evaluating the effectiveness of HIV-1 vaccines in preclinical animal models and humans. However, it is important to note that we lack data on autologous and heterologous tier-2 viruses' neutralization, which represent a limitation in our study as the induction of bnAbs is expected to be required for an effective HIV vaccine.

While rhesus macaques have been used for many HIV vaccine studies, we and others have extensively used cynomolgus macaques for assessing host immune response to SIV/SHIV infection and to HIV vaccines.[45–49] A direct comparison of both species should reveal if any of these models is more representative for human responses. However, most antibodies used to phenotype cluster determinants and to characterize cytokine production are anti-human determinant antibodies selected to cross-react with macaque determinants. As a consequence,

reagents availability represents a technical limitation that drives panel design, and some markers classically used to characterize cell populations in humans may not be available for NHPs. Furthermore, anti-human antibodies may result in slightly altered signal detection in flow and mass cytometry due to differences in binding affinity on macaque epitopes. Finally, markers expression may differ between species as some of them might be specific to a given species . Furthermore, a given marker expressed in both human and macaque may not be identified in the same cell population, thus limiting the full translation of the preclinical studies to the human situation.[50]

STAR★METHODS

Detailed methods are provided in the online version of this paper and include the following:

- KEY RESOURCES TABLE
- RESOURCE AVAILABILITY
  - Lead contact
  - Materials availability
  - Data and code availability
- EXPERIMENTAL MODEL AND SUBJECT DETAILS
  - Ethics and biosafety statement
  - Animals and study design
  - Adjuvants
- METHOD DETAILS
  - ELISA reagents and procedures
  - Neutralization assay
  - ELISA-based rsFcγRIIIa dimer-binding assay
  - Antibody coupling with lanthanide isotopes
  - Staining and CyTOF acquisition
  - Data processing
  - Cell population identification
  - Heatmap representation
  - Phenotypic and kinetic families
  - Negative-stain electron microscopy
- QUANTIFICATION AND STATISTICAL ANALYSIS
  - Statistics
  - Linear discriminant analysis
  - Cytocompare
  - Generalized linear model

SUPPLEMENTAL INFORMATION

Supplemental information can be found online at https://doi.org/10.1016/j.xcrm.2022.100751.

ACKNOWLEDGMENTS

We thank the staff of the animal facility of IDMIT, particularly B. Delache, M. Pottier, S. Langlois, J.M. Robert, N. Dhooge, and R. Ho Tsong Fang. We also thank S. Kent, M. Hogarth, and B. Wines for providing the dimeric FcγRIIIa protein. Dietmar Katinger provided the adjuvants. This study was supported by the European Union's Horizon 2020 (EAVI2020, grant N°681137), the European Infrastructure TRANSVAC2 (grant N° 730964), the Agence Nationale de Recherche sur le SIDA et les hépatites virales (ANRS) and the Bill and Melinda Gates Foundation (OPP1115782). R.W.S. is a recipient of a Vici fellowship from the Netherlands Organisation for Scientific Research (NWO). The Infectious Disease Models and Innovative Therapies Research Infrastructure (IDMIT) is supported by the "Programmes Investissements d'Avenir" (PIA), managed by the ANR under references ANR-11-INBS-0008 and ANR-10-EQPX-02-01. J.L.P. received a PhD scholarship from the University of Paris-Saclay. We thank the Fondation Dormeur Vaduz for the donation of instruments relevant to this project.

AUTHOR CONTRIBUTIONS

A.B.W. and O.G. and performed electron microscopy experiments. A.S.B. contributed to the mass cytometry experiments design. A.S.G. coordinated the cytometry platform and experiments. E.G. contributed to the data analysis. E.M.L. managed the mass cytometer and contributed to the results analysis. G.S. contributed to the design of the study and the analysis of the data and coordinated the neutralization assays. I.B. designed and produced the immunogens. J.L.P. designed and supervised the mass cytometry experiments and contributed to the data analysis. M.C. coordinated the FcgR-binding assays and contributed to analysis of the data. M.T. performed the neutralization assays and analysis. M.V.T. performed the mass cytometry experiments, analyzed the data, and wrote the article. N.D.B. managed the ethical authorizations, prepared the vaccines, and contributed to the study design, to coordination, and to the data analyses. P.M. contributed to the design of the study, coordinated the study and experiments, prepared the vaccines, and contributed to the data analysis. P.M.K. supervised the ELISA experiments. R.L.G. contributed to the study design and the results analysis. R.J.S. coordinated the EAVI2020 project and contributed to the study design and to the results analysis. R.S. contributed to the study design and coordinated the immunogens production. Y.A. performed and analyzed the ELISA. All authors reviewed and edited the manuscript.

DECLARATION OF INTERESTS

The authors declare no competing interests.

Received: September 24, 2021
Revised: June 9, 2022
Accepted: September 2, 2022
Published: September 26, 2022

REFERENCES

1. United Nations Joint Programme on HIV/AIDS (UNAIDS) (2019). UNAIDS Data 2019, p. 476.
2. Gaucher, D., Therrien, R., Kettaf, N., Angermann, B.R., Boucher, G., Filali-Mouhim, A., Moser, J.M., Mehta, R.S., Drake, D.R., 3rd, Castro, E., et al. (2008). Yellow fever vaccine induces integrated multilineage and polyfunctional immune responses. J. Exp. Med. *205*, 3119–3131.
3. Querec, T.D., Akondy, R.S., Lee, E.K., Cao, W., Nakaya, H.I., Teuwen, D., Pirani, A., Gernert, K., Deng, J., Marzolf, B., et al. (2009). Systems biology approach predicts immunogenicity of the yellow fever vaccine in humans. Nat. Immunol. *10*, 116–125.
4. Hou, J., Wang, S., Jia, M., Li, D., Liu, Y., Li, Z., Zhu, H., Xu, H., Sun, M., Lu, L., et al. (2017). A systems vaccinology approach reveals temporal transcriptomic changes of immune responses to the yellow fever 17D vaccine. J. Immunol. *199*, 1476–1489.
5. Rechtien, A., Richert, L., Lorenzo, H., Martrus, G., Hejblum, B., Dahlke, C., Kasonta, R., Zinser, M., Stubbe, H., Matschl, U., et al. (2017). Systems vaccinology identifies an early innate immune signature as a correlate of antibody responses to the ebola vaccine rVSV-ZEBOV. Cell Rep. *20*, 2251–2261.
6. Li, S., Rouphael, N., Duraisingham, S., Romero-Steiner, S., Presnell, S., Davis, C., Schmidt, D.S., Johnson, S.E., Milton, A., Rajam, G., et al. (2014). Molecular signatures of antibody responses derived from a systems biology study of five human vaccines. Nat. Immunol. *15*, 195–204.
7. Nakaya, H.I., Clutterbuck, E., Kazmin, D., Wang, L., Cortese, M., Bosinger, S.E., Patel, N.B., Zak, D.E., Aderem, A., Dong, T., et al. (2016). Systems

biology of immunity to MF59-adjuvanted versus nonadjuvanted trivalent seasonal influenza vaccines in early childhood. Proc. Natl. Acad. Sci. USA *113*, 1853–1858.

8. Tsang, J.S., Schwartzberg, P.L., Kotliarov, Y., Biancotto, A., Xie, Z., Germain, R.N., Wang, E., Olnes, M.J., Narayanan, M., Golding, H., et al. (2014). Global analyses of human immune variation reveal baseline predictors of postvaccination responses. Cell *157*, 499–513.
9. Trautmann, L., and Sekaly, R.P. (2011). Solving vaccine mysteries: a systems biology perspective. Nat. Immunol. *12*, 729–731.
10. Mooney, M., McWeeney, S., Canderan, G., and Sékaly, R.P. (2013). A systems framework for vaccine design. Curr. Opin. Immunol. *25*, 551–555.
11. Reimann, K.A., Parker, R.A., Seaman, M.S., Beaudry, K., Beddall, M., Peterson, L., Williams, K.C., Veazey, R.S., Montefiori, D.C., Mascola, J.R., et al. (2005). Pathogenicity of simian-human immunodeficiency virus SHIV-89.6P and SIVmac is attenuated in cynomolgus macaques and associated with early T-lymphocyte responses. J. Virol. *79*, 8878–8885.
12. Estes, J.D., Wong, S.W., and Brenchley, J.M. (2018). Nonhuman primate models of human viral infections. Nat. Rev. Immunol. *18*, 390–404.
13. Sanders, R.W., and Moore, J.P. (2017). Native-like Env trimers as a platform for HIV-1 vaccine design. Immunol. Rev. *275*, 161–182. https://doi.org/10.1111/imr.12481.
14. De Taeye, S.W., Ozorowski, G., de la Peña, A.T., Guttman, M., Julien, J.-P., van den Kerkhof, T.L.G.M., Burger, J.A., Pritchard, L.K., Pugach, P., Yasmeen, A., et al. (2015). Immunogenicity of stabilized HIV-1 envelope trimers with reduced exposure of non-neutralizing epitopes. Cell. https://doi.org/10.1016/j.cell.2015.11.056.
15. Sliepen, K., Han, B.W., Bontjer, I., Mooij, P., Garces, F., Behrens, A.J., Rantalainen, K., Kumar, S., Sarkar, A., Brouwer, P.J.M., et al. (2019). Structure and immunogenicity of a stabilized HIV-1 envelope trimer based on a group-M consensus sequence. Nat. Commun. *10*, 2355.
16. Brouwer, P.J.M., Antanasijevic, A., Berndsen, Z., Yasmeen, A., Fiala, B., Bijl, T.P.L., Bontjer, I., Bale, J.B., Sheffler, W., Allen, J.D., et al. (2019). Enhancing and shaping the immunogenicity of native-like HIV-1 envelope trimers with a two-component protein nanoparticle. Nat. Commun. *10*, 4272.
17. Wines, B.D., Vanderven, H.A., Esparon, S.E., Kristensen, A.B., Kent, S.J., and Hogarth, P.M. (2016). Dimeric FcγR ectodomains as probes of the Fc receptor function of anti-influenza virus IgG. J. Immunol. *197*, 1507–1516.
18. Zhang, L., Wang, W., and Wang, S. (2015). Effect of vaccine administration modality on immunogenicity and efficacy. Preprint at Expert Rev. Vaccines *14*, 1509–1523. https://doi.org/10.1586/14760584.2015.1081067.
19. Calabro, S., Tritto, E., Pezzotti, A., Taccone, M., Muzzi, A., Bertholet, S., De Gregorio, E., O'Hagan, D.T., Baudner, B., and Seubert, A. (2013). The adjuvant effect of MF59 is due to the oil-in-water emulsion formulation, none of the individual components induce a comparable adjuvant effect. Vaccine *31*, 3363–3369.
20. Pauthner, M., Havenar-Daughton, C., Sok, D., Nkolola, J.P., Bastidas, R., Boopathy, A.V., Carnathan, D.G., Chandrashekar, A., Cirelli, K.M., Cottrell, C.A., et al. (2017). Elicitation of robust tier 2 neutralizing antibody responses in nonhuman primates by HIV envelope trimer immunization using optimized approaches. Immunity *46*, 1073–1088.e6.
21. O'Hagan, D.T., Ott, G.S., De Gregorio, E., and Seubert, A. (2012). The mechanism of action of MF59 – an innately attractive adjuvant formulation. Vaccine *30*, 4341–4348.
22. Rosenbaum, P., Tchitchek, N., Joly, C., Rodriguez Pozo, A., Stimmer, L., Langlois, S., Hocini, H., Gosse, L., Pejoski, D., Cosma, A., et al. (2020). Vaccine inoculation route modulates early immunity and consequently antigen-specific immune response. SSRN J. https://doi.org/10.2139/ssrn.3535877.
23. Anderson, K.P., Lucas, C., Hanson, C.V., Londe, H.F., Izu, A., Gregory, T., Ammann, A., Berman, P.W., and Eichberg, J.W. (1989). Effect of dose and immunization schedule on immune response of baboons to recombinant glycoprotein 120 of HIV-1. J. Infect. Dis. *160*, 960–969.
24. Sanders, R.W., van Gils, M.J., Derking, R., Sok, D., Ketas, T.J., Burger, J.A., Ozorowski, G., Cupo, A., Simonich, C., Goo, L., et al. (2015). HIV-1 VACCINES. HIV-1 neutralizing antibodies induced by native-like envelope trimers. Science *349*, aac4223.
25. Brouwer, P.J.M., Antanasijevic, A., de Gast, M., Allen, J.D., Bijl, T.P.L., Yasmeen, A., Ravichandran, R., Burger, J.A., Ozorowski, G., Torres, J.L., et al. (2021). Immunofocusing and enhancing autologous Tier-2 HIV-1 neutralization by displaying Env trimers on two-component protein nanoparticles. NPJ Vaccines *6*, 24.
26. Schifanella, L., Barnett, S.W., Bissa, M., Galli, V., Doster, M.N., Vaccari, M., Tomaras, G.D., Shen, X., Phogat, S., Pal, R., et al. (2019). ALVAC-HIV B/C candidate HIV vaccine efficacy dependent on neutralization profile of challenge virus and adjuvant dose and type. PLoS Pathog. *15*, e1008121.
27. Vaccari, M., Fourati, S., Gordon, S.N., Brown, D.R., Bissa, M., Schifanella, L., Silva de Castro, I., Doster, M.N., Galli, V., Omsland, M., et al. (2018). HIV vaccine candidate activation of hypoxia and the inflammasome in CD14+ monocytes is associated with a decreased risk of SIVmac251 acquisition. Nat. Med. *24*, 847–856.
28. Mosca, F., Tritto, E., Muzzi, A., Monaci, E., Bagnoli, F., Iavarone, C., O'Hagan, D., Rappuoli, R., and De Gregorio, E. (2008). Molecular and cellular signatures of human vaccine adjuvants. Proc. Natl. Acad. Sci. USA *105*, 10501–10506.
29. Ols, S., Yang, L., Thompson, E.A., Pushparaj, P., Tran, K., Liang, F., Lin, A., Eriksson, B., Karlsson Hedestam, G.B., Wyatt, R.T., and Loré, K. (2020). Route of vaccine administration alters antigen trafficking but not innate or adaptive immunity. Cell Rep. *30*, 3964–3971.e7.
30. Lehmann, C.H.K., Heger, L., Heidkamp, G.F., Baranska, A., Lühr, J.J., Hoffmann, A., and Dudziak, D. (2016). Direct delivery of antigens to dendritic cells via antibodies specific for endocytic receptors as a promising strategy for future therapies. Vaccines *4*, 8.
31. Castro, F.V.V., Tutt, A.L., White, A.L., Teeling, J.L., James, S., French, R.R., and Glennie, M.J. (2008). CD11c provides an effective immunotarget for the generation of both CD4 and CD8 T cell responses. Eur. J. Immunol. *38*, 2263–2273.
32. White, A.L., Tutt, A.L., James, S., Wilkinson, K.A., Castro, F.V.V., Dixon, S.V., et al. (2010). Ligation of CD11c during vaccination promotes germinal centre induction and robust humoral responses without adjuvant. Immunology *131*, 141–151.
33. Zhang, Y., Boesen, C.C., Radaev, S., Brooks, A.G., Fridman, W.H., Sautes-Fridman, C., and Sun, P.D. (2000). Crystal structure of the extracellular domain of a human FcγRIII. Immunity *13*, 387–395.
34. Fromm, P., Papadimitrious, M., Hsu, J., Larsen, S.R., Gibson, J., Bradstock, K., Kupresanin, F., Clark, G., and Hart, D.N. (2016). CD16+ dendritic cells are a unique myeloid antigen presenting cell population. Blood *128*, 4897.
35. Schmitz, M., Zhao, S., Schäkel, K., Bornhäuser, M., Ockert, D., and Rieber, E.P. (2002). Native human blood dendritic cells as potent effectors in antibody-dependent cellular cytotoxicity. Blood *100*, 1502–1504.
36. Yeap, W.H., Wong, K.L., Shimasaki, N., Teo, E.C.Y., Quek, J.K.S., Yong, H.X., Diong, C.P., Bertoletti, A., Linn, Y.C., and Wong, S.C. (2016). CD16 is indispensable for antibodydependent cellular cytotoxicity by human monocytes. Sci. Rep. *6*, 34310–34322.
37. Palgen, J.L., Tchitchek, N., Huot, N., Elhmouzi-Younes, J., Lefebvre, C., Rosenbaum, P., Dereuddre-Bosquet, N., Martinon, F., Hocini, H., Cosma, A., et al. (2019). NK cell immune responses differ after prime and boost vaccination. J. Leukoc. Biol. *105*, 1055–1073.
38. Palgen, J.L., Tchitchek, N., Elhmouzi-Younes, J., Delandre, S., Namet, I., Rosenbaum, P., Dereuddre-Bosquet, N., Martinon, F., Cosma, A., Lévy, Y., et al. (2018). Prime and boost vaccination elicit a distinct innate myeloid cell immune response. Sci. Rep. *8*, 3087.
39. Palgen, J.L., Tchitchek, N., Rodriguez-Pozo, A., Jouhault, Q., Abdelhouahab, H., Dereuddre-Bosquet, N., Contreras, V., Martinon, F., Cosma, A.,

Lévy, Y., et al. (2020). Innate and secondary humoral responses are improved by increasing the time between MVA vaccine immunizations. NPJ Vaccines *5*, 24.

40. Andersen-Nissen, E., Fiore-Gartland, A., Ballweber Fleming, L., Carpp, L.N., Naidoo, A.F., Harper, M.S., Voillet, V., Grunenberg, N., Laher, F., Innes, C., et al. (2021). Innate immune signatures to a partially-efficacious HIV vaccine predict correlates of HIV-1 infection risk. PLoS Pathog. *17*, e1009363.
41. Gorini, G., Fourati, S., Vaccari, M., Rahman, M.A., Gordon, S.N., Brown, D.R., Law, L., Chang, J., Green, R., Barrenäs, F., et al. (2020). Engagement of monocytes, NK cells, and CD4+ Th1 cells by ALVAC-SIV vaccination results in a decreased risk of SIVmac251 vaginal acquisition. PLoS Pathog. *16*, e1008377.
42. Autissier, P., Soulas, C., Burdo, T.H., and Williams, K.C. (2010). Immunophenotyping of lymphocyte, monocyte and dendritic cell subsets in normal rhesus macaques by 12-color flow cytometry: clarification on DC heterogeneity. J. Immunol. Methods *360*, 119–128.
43. Sugimoto, C., Hasegawa, A., Saito, Y., Fukuyo, Y., Chiu, K.B., Cai, Y., Breed, M.W., Mori, K., Roy, C.J., Lackner, A.A., et al. (2015). Differentiation kinetics of blood monocytes and dendritic cells in macaques: insights to understanding human myeloid cell development. J. Immunol. *195*, 1774–1781.
44. Elhmouzi-Younes, J., Palgen, J.L., Tchitchek, N., Delandre, S., Namet, I., Bodinham, C.L., Pizzoferro, K., Lewis, D.J., Le Grand, R., Cosma, A., and Beignon, A.S. (2017). In depth comparative phenotyping of blood innate myeloid leukocytes from healthy humans and macaques using mass cytometry. Cytometry *91*, 969–982.
45. Moriarty, R.v., Rodgers, M.A., Ellis, A.L., Balgeman, A.J., Larson, E.C., Hopkins, F., Chase, M.R., Maiello, P., Fortune, S.M., Scanga, C.A., and O'Connor, S.L. (2022). Spontaneous control of SIV replication does not prevent T cell dysregulation and bacterial dissemination in animals Co-infected with M. tuberculosis. Microbiol. Spectr. *10*, e0172421.
46. Nzounza, P., Martin, G., Dereuddre-Bosquet, N., Najburg, V., Gosse, L., Ruffié, C., Combredet, C., Petitdemange, C., Souquère, S., Schlecht-Louf, G., et al. (2021). A recombinant measles virus vaccine strongly reduces SHIV viremia and virus reservoir establishment in macaques. NPJ Vaccines *6*, 123.
47. Cavarelli, M., Hua, S., Hantour, N., Tricot, S., Tchitchek, N., Gommet, C., Hocini, H., Chapon, C., Dereuddre-Bosquet, N., and Le Grand, R. (2021). Leukocytospermia induces intraepithelial recruitment of dendritic cells and increases SIV replication in colorectal tissue explants. Commun. Biol. *4*, 861.
48. le Grand, R., Dereuddre-Bosquet, N., Dispinseri, S., Gosse, L., Desjardins, D., Shen, X., Tolazzi, M., Ochsenbauer, C., Saidi, H., Tomaras, G., et al. (2016). Superior efficacy of a human immunodeficiency virus vaccine combined with antiretroviral prevention in simian-human immunodeficiency virus-challenged nonhuman primates. J. Virol. *90*, 5315–5328.
49. Lemaitre, J., Desjardins, D., Gallouët, A.S., Gomez-Pacheco, M., Bourgeois, C., Favier, B., Sáez-Cirión, A., Le Grand, R., and Lambotte, O. (2022). Expansion of immature neutrophils during SIV infection is associated with their capacity to modulate T-cell function. Front. Immunol. *13*, 781356.
50. Jacquelin, B., Mayau, V., Targat, B., Liovat, A.S., Kunkel, D., Petitjean, G., Dillies, M.A., Roques, P., Butor, C., Silvestri, G., et al. (2009). Nonpathogenic SIV infection of African green monkeys induces a strong but rapidly controlled type I IFN response. J. Clin. Invest. *119*, 3544–3555.
51. Vishwanathan, S.A., Burgener, A., Bosinger, S.E., Tharp, G.K., Guenthner, P.C., Patel, N.B., Birse, K., Hanson, D.L., Westmacott, G.R., Henning, T.R., et al. (2015). Cataloguing of potential HIV susceptibility factors during the menstrual cycle of pig-tailed macaques by using a systems biology approach. J. Virol. *89*, 9167–9177.
52. Han, Q., Bradley, T., Williams, W.B., Cain, D.W., Montefiori, D.C., Saunders, K.O., Parks, R.J., Edwards, R.W., Ferrari, G., Mueller, O., et al. (2020). Neonatal rhesus macaques have distinct immune cell transcriptional profiles following HIV envelope immunization. Cell Rep. *30*, 1553–1569.e6.
53. Heyndrickx, L., Heath, A., Sheik-Khalil, E., Alcami, J., Bongertz, V., Jansson, M., Malnati, M., Montefiori, D., Moog, C., Morris, L., et al. (2012). International network for comparison of HIV neutralization assays: the NeutNet report II. PLoS One *7*, e36438.
54. Suphaphiphat, K., Bernard-Stoecklin, S., Gommet, C., Delache, B., Dereuddre-Bosquet, N., Kent, S.J., Wines, B.D., Hogarth, P.M., Le Grand, R., and Cavarelli, M. (2020). Innate and adaptive anti-SIV responses in macaque semen: implications for infectivity and risk of transmission. Front. Immunol. *11*, 850.
55. Han, G., Spitzer, M.H., Bendall, S.C., Fantl, W.J., and Nolan, G.P. (2018). Metal-isotope-tagged monoclonal antibodies for high-dimensional mass cytometry. Nat. Protoc. *13*, 2121–2148.
56. Finck, R., Simonds, E.F., Jager, A., Krishnaswamy, S., Sachs, K., Fantl, W., Pe'er, D., Nolan, G.P., and Bendall, S.C. (2013). Normalization of mass cytometry data with bead standards. Cytometry A. *83*, 483–494.
57. Kleinsteuber, K., Corleis, B., Rashidi, N., Nchinda, N., Lisanti, A., Cho, J.L., Medoff, B.D., Kwon, D., and Walker, B.D. (2016). Standardization and quality control for high-dimensional mass cytometry studies of human samples. Cytometry A. *89*, 903–913.
58. Qiu, P., Simonds, E.F., Bendall, S.C., Gibbs, K.D., Jr., Bruggner, R.V., Linderman, M.D., Sachs, K., Nolan, G.P., and Plevritis, S.K. (2011). Extracting a cellular hierarchy from high-dimensional cytometry data with SPADE. Nat. Biotechnol. *29*, 886–891.
59. Gautreau, G., Pejoski, D., Le Grand, R., Cosma, A., Beignon, A.S., and Tchitchek, N. (2017). SPADEVizR: an R package for visualization, analysis and integration of SPADE results. Bioinformatics *33*, 779–781. https://doi.org/10.1093/bioinformatics/btw708.
60. Suloway, C., Pulokas, J., Fellmann, D., Cheng, A., Guerra, F., Quispe, J., Stagg, S., Potter, C.S., and Carragher, B. (2005). Automated molecular microscopy: the new Leginon system. J. Struct. Biol. *151*, 41–60.
61. Lander, G.C., Stagg, S.M., Voss, N.R., Cheng, A., Fellmann, D., Pulokas, J., Yoshioka, C., Irving, C., Mulder, A., Lau, P.W., et al. (2009). Appion: an integrated, database-driven pipeline to facilitate EM image processing. J. Struct. Biol. *166*, 95–102.
62. Pugach, P., Ozorowski, G., Cupo, A., Ringe, R., Yasmeen, A., de Val, N., Derking, R., Kim, H.J., Korzun, J., Golabek, M., et al. (2015). A native-like SOSIP.664 trimer based on an HIV-1 subtype B env gene. J. Virol. *89*, 3380–3395.
63. Sanders, R.W., Derking, R., Cupo, A., Julien, J.P., Yasmeen, A., de Val, N., Kim, H.J., Blattner, C., de la Peña, A.T., Korzun, J., et al. (2013). A next-generation cleaved, soluble HIV-1 env trimer, BG505 SOSIP.664 gp140, expresses multiple epitopes for broadly neutralizing but not non-neutralizing antibodies. PLoS Pathog. *9*, e1003618.
64. Platon, L., Pejoski, D., Gautreau, G., Targat, B., Le Grand, R., Beignon, A.S., and Tchitchek, N. (2018). A computational approach for phenotypic comparisons of cell populations in high-dimensional cytometry data. Methods *132*, 66–75.

STAR★METHODS

KEY RESOURCES TABLE

REAGENT or RESOURCE	SOURCE	IDENTIFIER
Antibodies		
CD66abce Antibody, anti-human	Miltenyi Biotec	Custom reagent (TET2)
Purified anti-human HLA-DR Antibody	Biolegend	Cat#307612; RRID:AB_314690
BD Pharmingen^{TM} Purified mouse anti-human CD3	BD Biosciences	Cat#551916; RRID:AB_394293
CD64 Antibody, anti-human	Miltenyi Biotec	Cat# 130-108-046; RRID:AB_2658939
BD Pharmingen^{TM} Purified mouse anti-human CD8	BD Biosciences	Cat# 555364; RRID:AB_395767
IL-6 Antibody, anti-human	Miltenyi Biotec	Cat#130-096-093; RRID:AB_2652449
BD Pharmingen^{TM} Purified Mouse Anti-Human CD123	BD Biosciences	Cat# 554527; RRID:AB_395455
BD Pharmingen^{TM} Purified Mouse Anti-Human IL-4	BD Biosciences	Cat# 554515; RRID:AB_398567
CD11a Antibody, anti-human	Miltenyi Biotec	Custom reagent (HI111)
BD Pharmingen^{TM} Purified Mouse Anti-Human CD11b	BD Biosciences	Cat# 555386; RRID:AB_395787
CD62L Antibody, anti-human	Miltenyi Biotec	Custom reagent (SK11)
BD Pharmingen^{TM} Purified Mouse Anti-Human CD4	BD Biosciences	Cat# 550625; RRID:AB_393787
Anti-human FceRI Prufied	eBioscience	14-5899-82
BD Pharmingen^{TM} Purified Mouse Anti-Human CD86	BD Biosciences	Cat# 555663; RRID:AB_396017
CD172a Antibody, anti-human	Miltenyi Biotec	Custom reagent (15–414/REA144 ; RRID:AB_2801909)
IP-10 Antibody, anti-human	Miltenyi Biotec	Cat#130-108-047 ; RRID:AB_2651479
BD Pharmingen^{TM} Purified Mouse Anti-Human CD45	BD Biosciences	Cat# 552566; RRID:AB_394433
IL-1a Antibody, anti-human	Miltenyi Biotec	Custom reagent (364-3B3–14)
Anti-hCD1c Affinity Purified Goat IgG	R&D systems	Cat# AF5910; RRID:AB_1964521
IL-12 Antibody, anti-human	Miltenyi Biotec	Custom reagent (C8.6 ; RRID:AB_10829623)
BD Pharmingen^{TM} Purified Mouse Anti-Human CD32	BD Biosciences	Cat# 555447; RRID:AB_395840
IFNa Antibody, anti-human	Miltenyi Biotec	130-108-050; RRID:AB_2659989
Purified anti-human CD39 (MaxPar® Ready)	Biolegend	Cat#328221; RRID:AB_2563747
BD Pharmingen^{TM} Purified Mouse Anti-Human CD195 (CCR5)	BD Biosciences	Cat# 556041; RRID:AB_396312
CD16 Antibody, anti-human	Miltenyi Biotec	130-108-027 ; RRID:AB_2655423
Purified anti-human CD11c (MaxPar® Ready)	Biolegend	Cat#301639; RRID:AB_2562812
BD Pharmingen^{TM} Purified Mouse Anti-Human CD184 (CXCR4)	BD Biosciences	Cat# 555972; RRID:AB_396265
BD Pharmingen^{TM} Purified Mouse Anti-Human CD14	BD Biosciences	Cat# 555396; RRID:AB_395797
BD Pharmingen^{TM} Purified Mouse Anti-Human IL-8	BD Biosciences	Cat# 554717; RRID:AB_398583
Mouse anti-human CD23	Beckman Coulter	IMBULK1
BD Pharmingen^{TM} Purified Mouse Anti-Human CD141	BD Biosciences	Cat# 559780; RRID:AB_397321
BD Pharmingen^{TM} Purified Mouse Anti-Human CD20	BD Biosciences	Cat# 556631; RRID:AB_396500
CCR7 Antibody, anti-human	Miltenyi Biotec	Custom reagent (G043H7)
Anti-SynCAM (TSLC1/CADM1)	MBL	Cat# CM004-3; RRID:AB_592783
Goat anti-Monkey IgG horseradish peroxidase labelled	AbSerotec	AAI42P
Biological samples		
Cynomolgus macaques PBMCs	IDMIT facility, CEA de Fontenay-aux-roses, France	N/A
Cynomolgus macaques serum	IDMIT facility, CEA de Fontenay-aux-roses, France	N/A

(Continued on next page)

Continued

REAGENT or RESOURCE	SOURCE	IDENTIFIER
Chemicals, peptides, and recombinant proteins		
Maxpar® X8 Antibody Labelling Kit, Tag-141Pr to 176Yb	Fluidigm	201141A to 201176A
Cell-ID™ Intercalator-Ir—500 μM	Fluidigm	201192B
Cell-ID™ Intercalator-Rh—2000 μM	Fluidigm	201103B
ConM SOSIP.v7 protein	Rogier Sanders (Sliepen et al., 2019)	https://doi.org/10.1038/s41467-019-10262-5
Mono Phosphoryl Lipid A (MPLA adjuvant)	Polymun Scientific (Klosterneuburg, Austria)	N/A
Squalen Emulsion (SQ adjuvant)	Polymun Scientific (Klosterneuburg, Austria)	N/A
BSA cat#A7906-1kg	Sigma Aldrich	SLBS4333
Purified dimeric macaque rsFcγRIIIa Ile158 ectodomain biotin	Hogarth laboratory	N/A
Ultra streptavidin HRP	Thermo Scientific	cat# N504
TMB	Thermo Scientific	cat# 34028-250mL
HCL 1N	VWR	MC3006470500
EDTA	Sigma Aldrich	cat# E5391-250g
Tween 20	Sigma Aldrich	cat# P7949-100mL
SOSIP antigens	Rogier Sanders Amsterdam University Medical Centers, Amsterdam Netherlands	N/A
Bright-Glo	Promega	Cat# E2620
Experimental models: Cell lines		
HEK293F cells	Invitrogen	R79009
HEK-293T/17	CFAR-NIBSC, UK	Cat# 5016
TZM-bl	CFAR-NIBSC, UK	Cat#ARP5011
Experimental models: Organisms/strains		
Cynomolgus macaques: *Macaca fascicularis*	AAALAC certified breeding centers	N/A
Recombinant DNA		
HIV-1 backbone pSG3 ΔEnv	David C. Montefiori Duke Human Vaccine Institute and Center for HIV-AIDS Vaccine Immunology, Duke University Medical Center, Durham, NC,USA	N/A
pSVIII-93MW 965.26	CFAR-NIBSC, UK	Cat# 2073
pConM SOSIP.v7 HIV-1	Rogier Sanders Amsterdam University Medical Centers, Amsterdam Netherlands	N/A
Software and algorithms		
MatLab compiler software	Beckman Coulter	N/A
CyTOF software	Fluidigm	N/A
Cytobank Premium	Beckman Coulter	N/A
R software		N/A
SPADEVizR R package	https://github.com/tchitchek-lab/SPADEVizR	N/A
“MASS” R package	https://CRAN.R-project.org/package=MASS	N/A
Leginon	(Suloway et al., 2005)	https://nramm.nysbc.org/software/; RRID:SCR_016731
Appion	(Lander et al., 2009)	https://nramm.nysbc.org/software/; RRID:SCR_016734

(*Continued on next page*)

Continued		
REAGENT or RESOURCE	SOURCE	IDENTIFIER
Other		
F96 Nunc maxisorp plate	Scientific laboratory supplies	# 442404
Infectious molecular clone (IMC) ConM	Rogier Sanders (Sliepen et al., 2019)	https://doi.org/10.1038/s41467-019-10262-5
Mithras luminometer	Berthold Italia S.r.l	N/A

RESOURCE AVAILABILITY

Lead contact

Correspondence and requests for materials should be addressed to Roger Le Grand (roger.le-grand@cea.fr).

Materials availability

This study did not generate new unique reagents. Requests for materials will require specific agreements and should be address to the lead contact, Roger Le Grand.

Data and code availability

The data that support the findings of this study are available from the corresponding author upon reasonable request. This paper does not report original code. Any additional information required to reanalyze the data reported in this work paper is available from the lead contact upon request.

EXPERIMENTAL MODEL AND SUBJECT DETAILS

Ethics and biosafety statement

Cynomolgus macaques (*Macaca fascicularis*) originating from Mauritius and imported from AAALAC certified breeding centers were used in this study. All animals were housed in groups at the IDMIT infrastructure facilities (CEA, Fontenay-aux-roses, Animal facility authorization #D92-032-02, Prefecture des Hauts de Seine, France) and in compliance with European Directive 2010/63/EU, the French regulations, and the Standards for Humane Care and Use of Laboratory Animals, of the Office for Laboratory Animal Welfare (OLAW, assurance number #A5826-01, US). The protocols were approved by the institutional ethical committee "Comité d'Ethique en Expérimentation Animale du Commissariat à l'Energie Atomique et aux Energies Alternatives » (CEtEA #44) under statement number A15-073. The study was authorized by the "Research, Innovation and Education Ministry" under registration number APAFIS#3132–2015121014521340.

Animals and study design

Eighteen adult, female cynomolgus macaques, aged 33 to 42 months, were randomly assigned to three experimental groups of six animals each. All animals were immunized at W0, W8, and W24 with 20 μg ConM SOSIP formulated either with 500 μg MPLA liposomes (MPLA) or 0.5 mL squalene emulsion (SQ). The vaccine was divided between each thigh. One group was immunized by the subcutaneous (SC) route with the MPLA regimen (MPLA-SC), one received the MPLA regimen by the intramuscular (IM) route (MPLA-IM), and the third group received the SQ by the IM route (SQ-IM).

Group size of animals has been defined accordingly to the primary end-point of the study aiming at identifying the best combination of route and adjuvant for generating neutralizing antibodies when comparing the means of the experimental groups. A level of significance of 5% ($\alpha = 0.05$) and a target power of 80% ($1 - \beta = 0.8$) was established. As a consequence, the system biology approach we proposed for identification of early cellular markers predicting Nab response, was exploratory and the N was not defined accordingly to this secondary objective. However, the size of our experimental groups is in the range of similar previously published studies using NHP.[50–52]

Animals were observed daily, and clinical exams were performed at baseline and at each bleeding, as described in Figure 1, on anesthetized animals using ketamine (5 mg.kg-1) and metedomidine (0.042 mg.kg-1). Body weight and rectal temperature were recorded, and blood was collected. Blood cell counts, hemoglobin levels, and the hematocrit were determined from EDTA blood using a HMX A/L analyzer (Beckman Coulter). *ConM SOSIP.v7*.

ConM SOSIP.v7 protein was expressed and purified as published previously.[15,16] In brief, ConM SOSIP.v7 was expressed in transiently transfected HEK293F cells (Invitrogen, catalog number R79009) and purified from vacuum-filtered (0.2 μm filters) transfection supernatants by PGT145 bNAb-affinity chromatography. The protein was verified by SDS-PAGE analysis and Blue native PAGE analysis as described.[15,16]

Adjuvants

The MPLA liposomes and squalene emulsion were manufactured at Polymun Scientific (Klosterneuburg, Austria). MPLA batch number MPLA/L02/0.2 μm-filtered was mixed extemporaneously at 500 μg/mL with 20 μg SOSIP in PBS to 1 mL. Squalene emulsion batch IMP/300916/10.22-μm filtered was composed of an oil phase (5% v/v squalene and 0.5% w/v Span 85) in an aqueous phase (0.5% w/v Tween 80) and 0.5 mL was mixed with 20 μg SOSIP extemporaneously in PBS to 1 mL.

METHOD DETAILS

ELISA reagents and procedures

Antigen-specific IgG levels were assessed by capture ELISA using Myc-c tagged ConM SOSIP.664 v7 for capture by monoclonal Ab 9E10 (ATCC hybridoma). Nunc MaxiSorp high binding 96-well plates (Thermo Fisher Scientific) were coated with 2.5 μg/mL of 9E10 Ab in 100 μL/well 1× PBS. The last 3 columns of each plate were coated with goat anti-human Kappa and goat anti-human Lambda (Southern Biotech) for the captured standard IgG, 1:2,000 for each antibody in 100 μL/well 1× PBS. The plates were incubated overnight at +4°C, washed 4 times with 350 μL/well 1× PBS-0.05% Tween 20, tapped dry and blocked 1 h at +37°C with 200 μL/well casein buffer (Thermo Fisher Scientific). Following 4 washes, 1 μg/mL ConM SOSIP.664 v7 Myc-HIS trimers in 100 μL/well casein buffer were loaded onto the plates and standard wells with 100 μL/well CB without tagged protein. After a 1 h incubation at +37°C, plates were washed again and diluted samples added in triplicate wells in 50 μL/well casein buffer (1:100, 1:1,000, 1:10,000 dilutions). The cynomolgus macaque IgG standard (Molecular Innovations) was serially diluted in casein buffer starting at 200 ng/mL (1:5 serial dilution) and then loaded onto the standard wells (50 μL/well). The plates were incubated at +37°C for 1 h, washed and mouse anti-rhesus monkey IgG Fc biotinylated Ab (Southern Biotech) loaded using a 1:50,000 dilution in 100 μL/well casein buffer. Following another hour incubation at +37°C and washing step, poly-HRP40 was loaded onto the wells at 1:10,000 dilution in casein buffer (100 μL/well). Finally, after 1 h at +37°C, the plates were washed, tapped dry and developed for 5 min with 50 μL/well Sureblue TMB substrate and stopped with 50 μL/well Stop solution (KPL). A KC4 Spectrophotometer (BioTek) was used to read the absorbance at 450 nm and a 4-PL fit curve was used to determine the standard curves and the linear range where the appropriate sample dilution would fall into. Standard curves and raw data were interpolated in the SoftMax Pro software (Molecular Devices), exported as text files, analysed in Excel (Microsoft) and plotted in Prism v7.0 (GraphPad Software).

Neutralization assay

Env-pseudotyped virus (PSV) 93MW965.26 and infectious molecular clone (IMC) ConM[15] were produced in HEK293T cells, tittered and used in TZM-bl assay to determine nAb responses as previously described.[53] Briefly, duplicates of six steps of 3-fold dilution, starting with 1:20 of each serum, were incubated with viral supernatant (at relative luminescence units (RLU) between 150,000 and 200,000) for 1 h. Thereafter, 10^4 TZM-bl cells were added, and plates incubated for 48 h at 37°C, when Bright-Glo Luciferase assay system (Promega, Madison, Wisconsin, USA) was added to measure luciferase activity with a Mithras luminometer (Berthold, Germany). Positive controls were sera of HIV-1-infected individuals and monoclonal antibodies with known neutralizing titers. Testing against VSV was used to exclude unspecific reactions. Neutralization titers were defined as the sample dilution at which RLU were reduced by 50% compared to virus control wells after subtraction of background RLU in control wells with only cells. Inhibitory concentrations 50 (IC50) were calculated with a linear interpolation method using the mean of the duplicate responses.[53]

ELISA-based rsFcγRIIIa dimer-binding assay

The protocol of the ELISA-based IgG assay using recombinant soluble FcγRIIIa dimer has been previously described.[17,54] Briefly, ELISA plates (MaxiSorp plates; Nalgene Nunc, Rochester, NY) were coated overnight at 4°C with ConM SOSIP.v7 protein diluted to 50 ng per well in PBS, as well as no Ag as a negative control. HIVIG (#3957; National Institutes of Health AIDS Reagent) was used at 5 μg/mL to normalize the results across all plates. Coated plates were subsequently washed with PBS containing 0.05% Tween 20 (Sigma Aldrich) (PBST) and blocked with blocking buffer, consisting of PBS containing 1 mM EDTA and 1% BSA (both from Sigma- Aldrich), for 1 h at 37°C. Macaque sera were diluted 1/50 in blocking buffer and incubated for 1 h at 37°C. After washing 5 times the plates with PBST, purified dimeric macaque rsFcγRIIIa-biotin (I158 allele) was added to the plate at a concentration of 0.1 μg/mL and the plates incubated for 1 h at 37°C.Plates were washed again 5 times with PBST, before horseradish peroxidase (HRP)-conjugated streptavidin (Thermo Fisher Scientific) was added and the plates incubated for another 1 h at 37°C. After washing, the color was developed using 3,3′, 5,5-tetramethylbenzidine (TMB) (Life Technologies), followed by the addition of 1 M HCl stop solution. The absorbance at a wavelength of 450 nm was recorded. The no-Ag values were subtracted from each Ag sample. The resulting absorbance values were multiplied by the serum dilution factor. A positive signal was defined as an absorbance higher than the mean +3 x SD of the one obtained using sera from macaques before immunization.

Antibody coupling with lanthanide isotopes

Four hundred micrograms of each antibody was conjugated to metals using MaxPar X8 conjugation kits (DVS Science) following the manufacturer's instructions. Briefly, lanthanides were loaded onto the polymer and then purified by centrifugation through an AMICON 3-kDa filter (Merck Millipore). Antibodies were also purified using an AMICON 50-kDa filter and partially reduced by TCEP-R-Buffer before conjugation to the metal-loaded polymers.[55] Following conjugation, antibodies were resuspended at

1 μg/μL in Candor PBS Antibody Stabilization solution (Candor Bioscience) with 0.05% sodium azide and stored at 4°C. All conjugated monoclonal Ab used for labeling are shown in Table S3. The same batch of Ab were used for the different timepoints.

Staining and CyTOF acquisition

Whole blood was collected in heparin/lithium tubes and cryopreserved in 10% FCS/DMSO. Samples were rapidly thawed, washed with RPMI medium, and then incubated in RPMI supplemented with 10 mg/mL DNAse for 30 min at 37°C. Cells were then resuspended in 1× PBS and incubated for 15 min at 37°C with 1 μL Intercalator-Rh (DVS Sciences). After washing in PBS-0.5% BSA, cells were stained for 30 min at 4°C, washed with 1× PBS, fixed with PBS-1.6% PFA, and washed with 1× Perm/Wash Buffer (eBiosciences). After permeabilization with 1× Perm/Wash (eBiosciences), intracellular staining was performed for 30 min at 4°C. Cells were washed, fixed in 1.6% PFA, and washed with Barcode Perm Buffer (DVS) before barcoding with a unique combination of three palladium isotopes (DVS, Fluidigm) according to the manufacturer's recommendations. Cells were then washed with PBS and resuspended in PBS-1.6% PFA containing 0.5 μL Intercalator-Ir (DVS Sciences). Barcoded samples were pooled and stored overnight at 4°C. Before acquisition, cells were washed with milli-Q water and filtered through a cap filter with 35-μm pores (BD Biosciences). Normalization beads (Eq Beads, Fluidigm) were added to the tube and the acquisition was performed using a Helios mass cytometer (Fluidigm). Data were acquired for six consecutive days (3 animals per day).

Data processing

MatLab compiler software[56] was used on FCS files to compensate for signal loss by the detector during acquisition based on the signal given by the Eq Beads. Normalized data from each acquisition were concatenated (Cytobank concatenation tool) and all conditions separated according to their barcode signal using CyTOF software (Fluidigm). In addition, as we previously describe, we used the same sample for each experiment to control the quality of each staining/acquisition and thus its reproducibility.[57] Eq beads, dead cells, and cell doublets were excluded from the resulting FCS files, as well as nonspecifically stained $CD66^{+}CD3^{+}$ eosinophils, as previously described.[44]

Cell population identification

The spanning-tree progression analyses of density-normalized events algorithm (SPADE)[58] was used on the entire data set of macaque samples to cluster cell populations displaying similar phenotypes. The following markers were used for clustering: CD66, HLA-DR, CD3, CD64, CD8, CD123, CD11a, CD11b, CD62L, CD4, FcεRI, CD86, CD172a, CD45, CD1c, CD32, CD39, CCR5, CD16, CD11c, CXCR4, CD14, CD23, CD141, CD20, CCR7, and CADM1. A random pre-downsampling of 11,478 cells (corresponding to the number of cells contained in the smallest sample) was performed on all samples to allow an equal contribution of each sample to create the tree of cell clusters. All cells from every sample were then assigned to a cluster according to their phenotype.

The SPADEVizR R package[59] was used to perform quality control on the SPADE clusters. Clusters of good quality were defined based on a narrow (IQR $\leq$2) and unimodal distribution (Hartigan's dip test, $p \leq 0.05$) for each marker. After benchmarking of SPADE parameters, the optimal SPADE used 800 clusters and a downsampling limit of 30%, resulting in 64.62% of clusters of good quality.

Heatmap representation

A heatmap representation of the clusters was generated using SPADEVizR according to the mean median MSI of each sample, in which they were divided into five categories between the 5th and 95th percentile. For each cluster, samples contributing <10 cells were excluded. Hierarchical clustering of cell clusters and markers was performed using the Euclidean metric based on the ward.D linkage.

Phenotypic and kinetic families

We created phenotypic families, which grouped cell clusters that displayed similar phenotypes. This strategy avoids "over-clustering", as clusters may account for diverse activation or maturation stages, and favors the identification of actual subpopulations by their phenotypic characteristics. The phenotypic families (PF) were grouped accordingly to their evolution at the different time points of the study. We thus grouped the PF with similar evolution trends in "kinetics families" (KF). The grouping of PF to form KF was performed using a hierarchical method based on the Pearson correlation and complete linkage.

Negative-stain electron microscopy

Trimer and adjuvants were co-formulated as described above (see "Adjuvants") using the same ratios, scaled down 10-fold (i.e. total volume 0.1 mL). The formulations were incubated for 1 h at 37°C and diluted 1:10 in Tris-buffered saline before applying 3 μL onto glow-discharged carbon-coated Cu400 mesh grids (Electron Microscopy Services). The samples were blotted using filter paper and the grids were then negatively stained with 2% (w/v) uranyl formate for 60 s. Data were collected on a Tecnai Spirit transmission electron microscope, operating at 120 keV. Nominal magnification was 52,000× with a resulting pixel size of 2.05 Å at the specimen plane, and an average defocus of −1.50 μm was used. Micrographs were recorded using a Tietz 4k x 4k TemCam-F416 CMOS camera. Data collection was performed using Leginon automated imaging interface,[60] and data processing (particle picking, extraction and 2D classifications) was performed using the Appion data processing suite.[61] Data processing procedures to determine amount of native-like trimers is described in detail elsewhere.[62] Briefly, 2D class averages were visually inspected. Trimers visually similar to

those previously described for BG505 SOSIP.664[63] or B41 SOSIP.664[62] were considered to have an overall native structure. Any particles that did not clearly show a central, triangular mass were classified as non-native.

QUANTIFICATION AND STATISTICAL ANALYSIS

Statistics

R software was employed to perform SPADE algorithm and statistical analysis related to mass cytometry was performed using SPADEVizR (available at https://github.com/tchitchek-lab/SPADEVizR) and "MASS" R package (available at https://CRAN.R-project.org/package=MASS). Comparisons of cell abundances were performed using the Mann-Whitney-Wilcoxon test, with p-values < 0.05 considered significant. No correction was applied because limited number of animals (n = 6) per groups. Because of this limitation, our intention was to use this approach to generate hypothesis rather than generalized observations. Thus, we tolerate a part of false-discovery rate in order to not exclude false negative data. Furthermore, as we performed a longitudinal follow up on the same macaque over time, comparisons are not independent to each other, so this would likely over-correct p-values.

Linear discriminant analysis

Linear discriminant analysis was performed using mean of non-standardized cell abundances of all individuals, to preserve the contribution of each variable and to differentiate vaccine regimens for each timepoint and identify KF associated to the different vaccines. In Figure 5A, the mean KF abundances of all individual was used as entry parameters to differentiate vaccine regimens at the different timepoint. In Figure 5B, mean KF abundances for all individuals and all timepoint were employed to identify the contribution of each KF to the different vaccine regimens discrimination.

Cytocompare

Distributions of marker expression were compared using the CytoCompare R package[64] to identify the contribution of each marker in the discrimination of vaccine signature. This R package use the Kolmogorov Smirnov distance to assess the maximum distance between cumulative distribution function (CDF) of the different markers based on their median signal intensity (MSI) for dendritic cells and monocytes. Then, as we aimed to prioritize the identification of a top discriminant markers (although, all markers could be statistically discriminant), we determined the threshold of 0.5, rather than Selecting a top set of markers in each comparison with the highest KS distance as we previously reported.[37]

Generalized linear model

A generalized linear model was used to assess the link between innate and adaptive responses after the two boosts. SPADEVizR R package is able to generate generalized linear models (GLM) to predict biological outcome associated to each sample based on cluster abundances. This method aims to identify a linear combination of clusters abundances that correlate with a biological outcomes from a training dataset. Based on these linear combinations, we can then predict biological outcomes for a test dataset. The abundance profiles of kinetic families for each individual 24h after the second and third injection (n = 12 for each vaccine regimen) were used as the entry parameter. The values to predict were the nAb titers, IgG binding titers and rsFcgRIIIa binding activities. Abundances 24h after the baseline were not included as no nAb were detected for this timepoint. Then, iterative linear regressions were generated until all coefficients had a p -value $\leq$0.05. At each iteration, the coefficient having the highest p-value higher than 0.05 was removed. One value was excluded from both the IgG binding and nAb model because titers at W26 for one animal were not included in the confidence interval.

Cell Reports Medicine, Volume *3*

Supplemental information

Innate cell markers that predict anti-HIV neutralizing antibody titers in vaccinated macaques

Matthieu Van Tilbeurgh, Pauline Maisonnasse, Jean-Louis Palgen, Monica Tolazzi, Yoann Aldon, Nathalie Dereuddre-Bosquet, Mariangela Cavarelli, Anne-Sophie Beignon, Ernesto Marcos-Lopez, Anne-Sophie Gallouet, Emmanuel Gilson, Gabriel Ozorowski, Andrew B. Ward, Ilja Bontjer, Paul F. McKay, Robin J. Shattock, Gabriella Scarlatti, Rogier W. Sanders, and Roger Le Grand

Innate cell markers that predict anti-HIV neutralizing antibody titers in vaccinated macaques

Supplementary materials

Authors: Matthieu Van Tilbeurgh[1], Pauline Maisonnasse[1], Jean-Louis Palgen[1], Monica Tolazzi[2], Yoann Aldon[3], Nathalie Dereuddre-Bosquet[1], Mariangela Cavarelli[1], Anne-Sophie Beignon[1], Ernesto Marcos-Lopez[1], Anne-Sophie Gallouet[1], Emmanuel Gilson[4], Gabriel Ozorowski[5], Andrew B. Ward[5], Ilja Bontjer[6], Paul F. McKay[3], Robin J. Shattock[3], Gabriella Scarlatti[2], Rogier W. Sanders[6,7], and Roger Le Grand[1]*.

Affiliations:

[1] Université Paris-Saclay, Inserm, CEA, Center for Immunology of Viral, Auto-immune, Hematological and Bacterial diseases » (IMVA-HB/IDMIT), 92265 Fontenay-aux-Roses & 94270 Le Kremlin-Bicêtre, France

[2] Viral Evolution and Transmission Unit, Division of Immunology, Transplantation and Infectious Diseases, IRCCS Ospedale San Raffaele, 20132 Milan, Italy

[3] Imperial College London, Faculty of Medicine, Department of Infectious Disease, London, United Kingdom.

[4] Life & Soft, 28 rue de la Redoute, 92260 Fontenay-aux-Roses, France

[5] Department of Integrative Structural and Computational Biology, The Scripps Research Institute, La Jolla, CA 92037, USA.

[6] Department of Medical Microbiology and Infection Prevention, Amsterdam University Medical Centers, Location AMC, University of Amsterdam, 1105 AZ Amsterdam, the Netherlands

[7] Department of Microbiology and Immunology, Weill Medical College of Cornell University, New York, NY 10021, USA

* Corresponding author: roger.le-grand@cea.fr

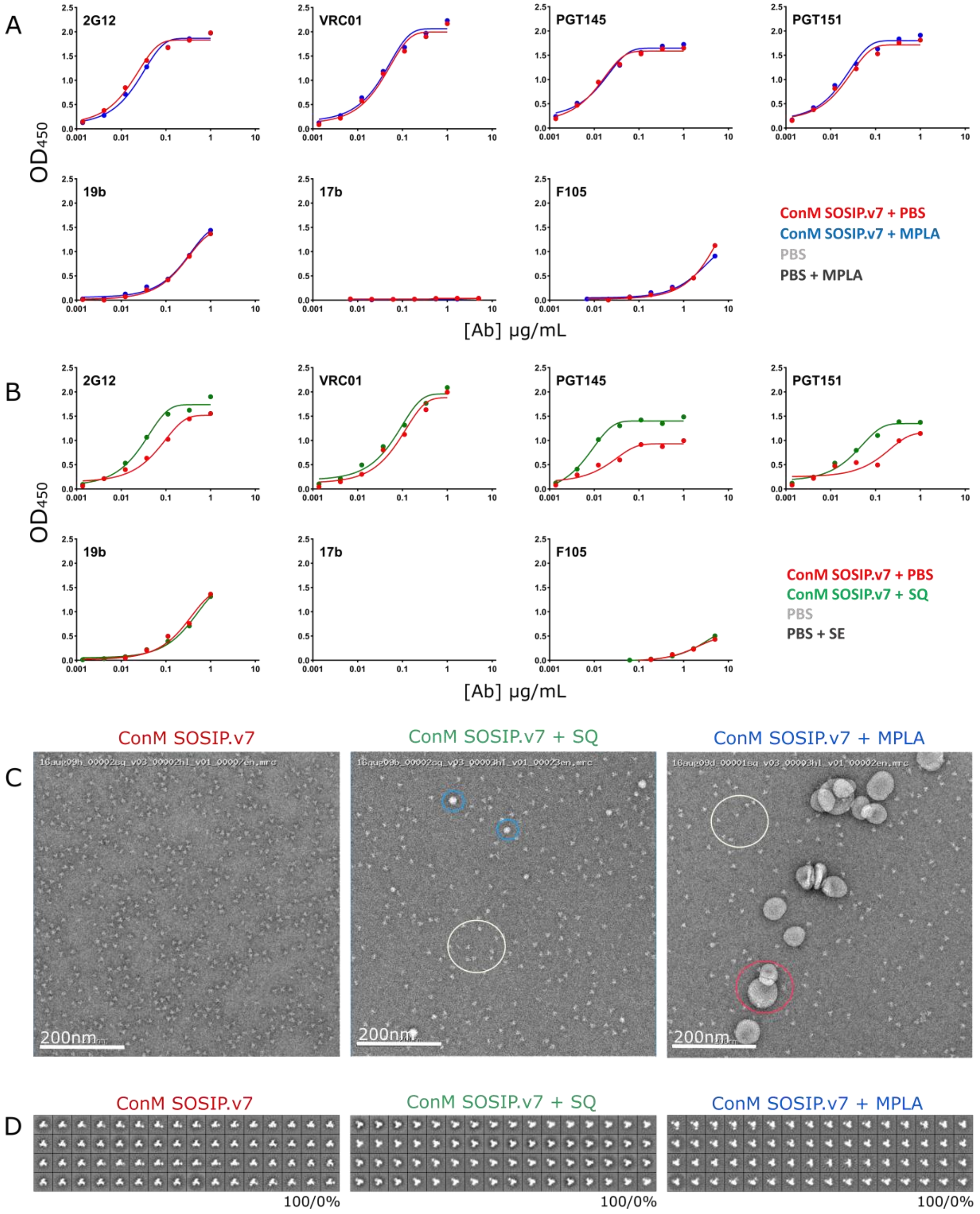

Figure S1. Formulation of ConM SOSIP.v7 with MPLA and SQ adjuvants (related to figure 2). D7324-tagged ConM SOSIP.v7 was incubated with the corresponding adjuvant for 1 h at 37ºC and the antigenicity (**A**, **B**) and morphology (**C**, **D**) were subsequently assessed by D7324-capture ELISA and negative-stain electron microscopy, respectively. (**C**) Electron micrographs of ConM SOSIP.v7 plus adjuvants; White, blue and red circles identified respectively trimers, Squalene and MPLA. (**D**) 2D-class averages of ConM SOSIP.v7 proteins in formulations. Percentage of native-like/non-native trimers (see *Materials and Methods*) are listed below each set of class averages.

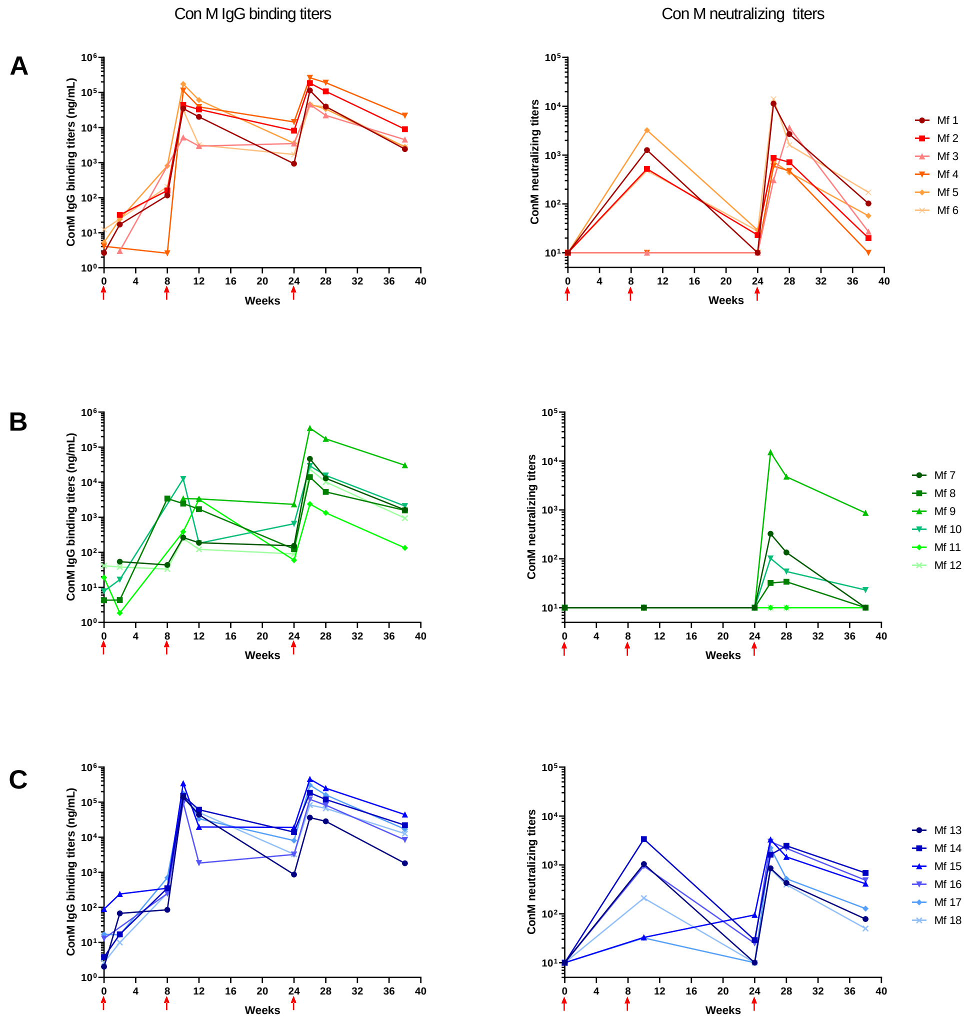

Figure S2. Individual ConM SOSIP v7 humoral responses (related to figure 2). ConM IgG binding and neutralizing titers of cynomolgus macaques from the MPLA-IM (A), MPLA-SC (B), and SQ-IM (C) groups, with red, green, and blue shading, respectively. Immunizations (week 0, 8, and 24) are indicated by the red arrows.

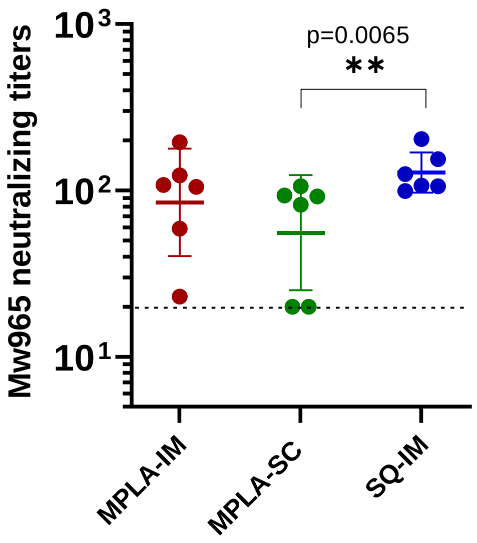

Figure S3: Heterologous neutralization against MW965 virus (related to figure 2). MW965 neutralizing titers were measured at week (W)28. Animals from the MPLA-IM group are displayed in red, the MPLA-SC group in green, and the SQ-IM group in blue. The geometric mean and geometric SD are represented. Comparison realized with a Mann-Whitney-Wilcoxon test with p.values < 0.05 are displayed.

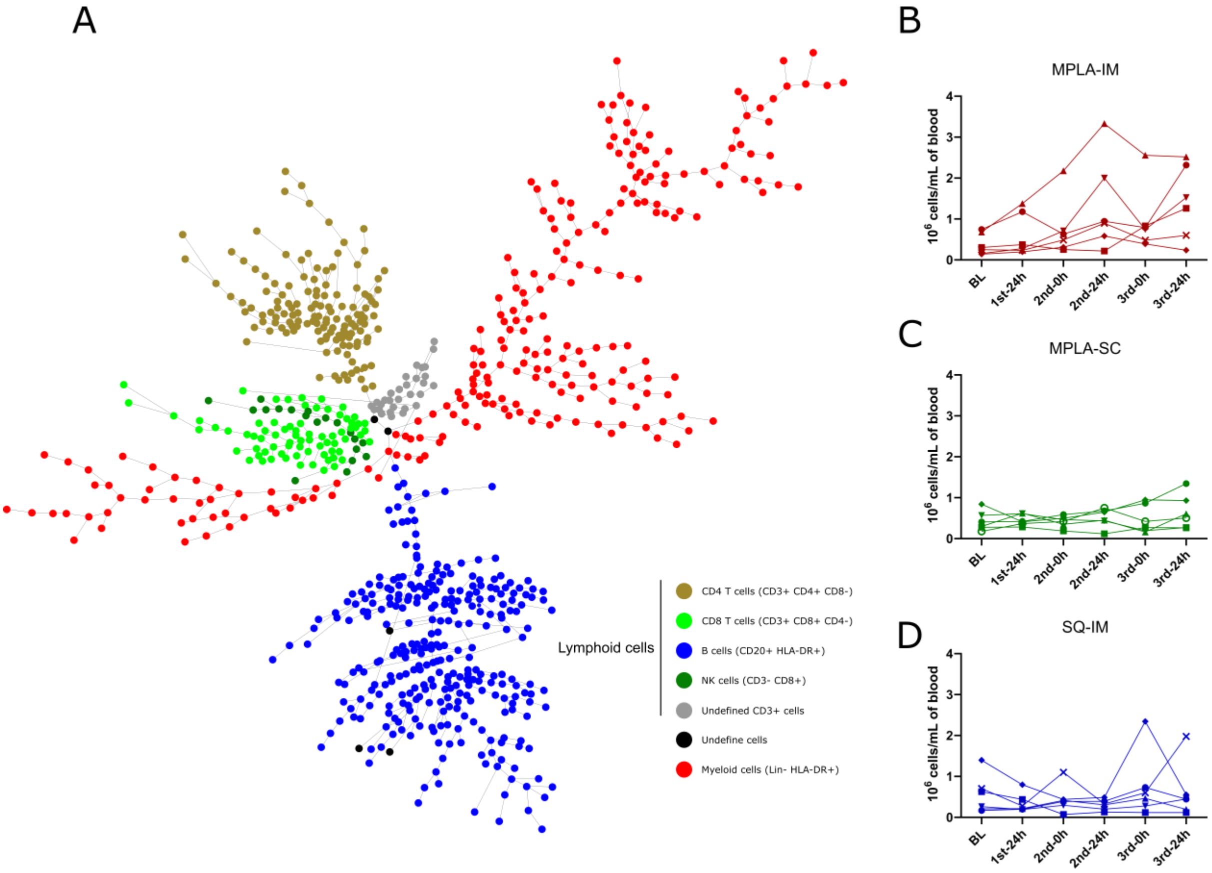

Figure S4. Identification of major cell populations using the SPADE clustering method following immunizations with SOSIP v7 ConM (related to figure 3). (**A**) SPADE tree created using the entire mass cytometry data set. Each dot represents a cell cluster. Major cell populations are annotated according to their phenotype: B cells ($CD20^+$ $HLA\text{-}DR^+$ $CD1c^+$), T cells ($CD3^+$ $CD4^+$ or $CD8^+$), NK cells ($CD3^-$ $CD8^+$), undefined lymphocytes ($CD3^+$ $CD4^-$ $CD8^{low/-}$), and undefined cells. All remaining cell clusters are considered to be myeloid cells. Phenotypically similar clusters were linked using a minimal spanning-tree approach of the SPADE algorithm. Kinetics of the myeloid cells compartment depending on the vaccine: (**B**) MPLA-IM, (**C**) MPLA-SC and (**D**) SQ-IM.

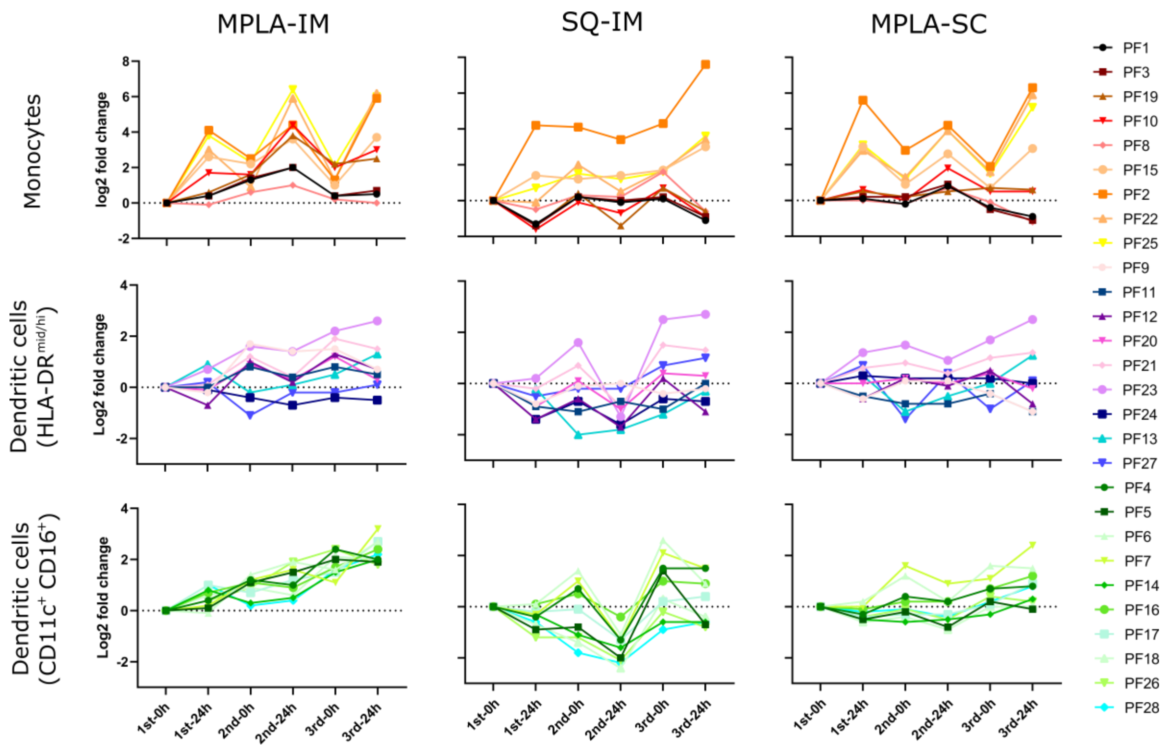

Figure S5. Variation of phenotypic families following immunization (related to figure 4). Mean log2 fold changes relative to the baseline values are displayed for each superfamily. Each color is assigned to a phenotypic family according to the colors defined in the heatmap (Fig.3).

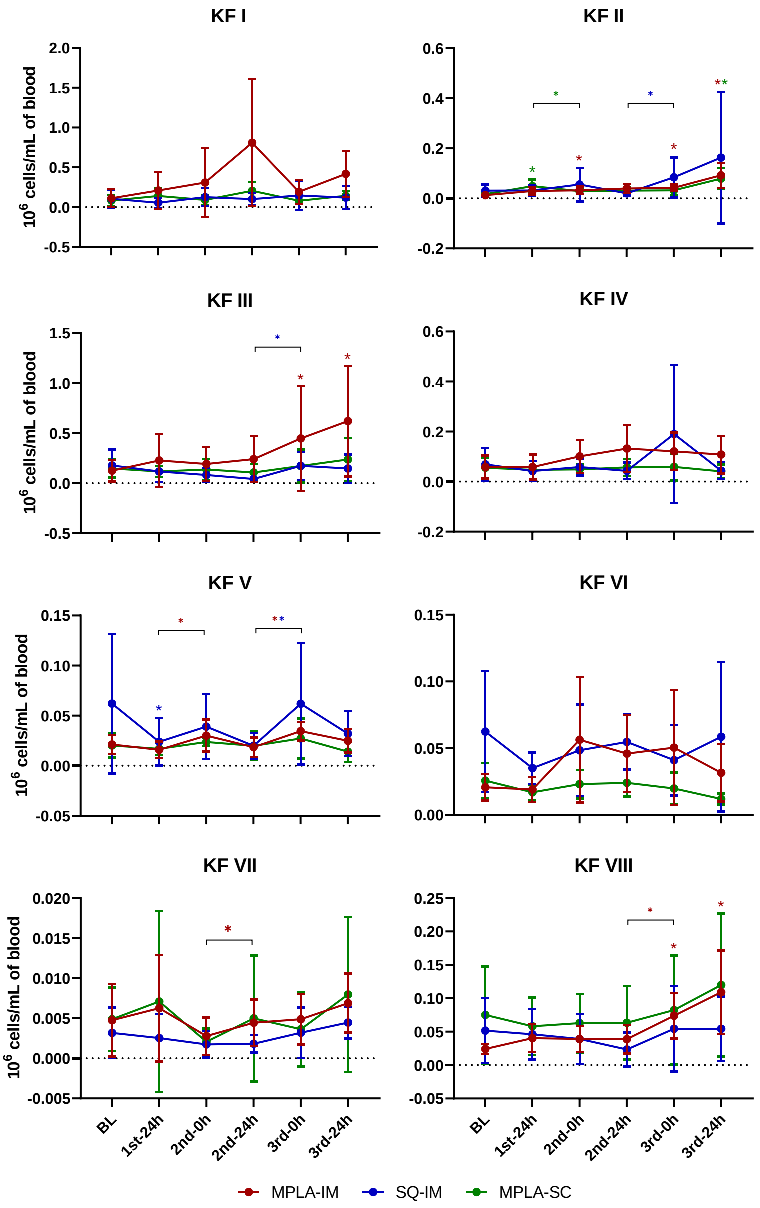

Figure S6. Dynamics of kinetic families following vaccine injections (related to figure 5). Phenotypic families were regrouped by similar dynamics to form eight kinetic families. The graphs show the mean cell number per mL of blood for all individuals (n=6) for each injection (MPLA-IM in red, MPLA-SC in green, and SQ-IM in blue). Significant differences relative to baseline values are indicated by a star and black lines are used to show significant differences compared to the previous time-point. P-values are shown in Table S2.

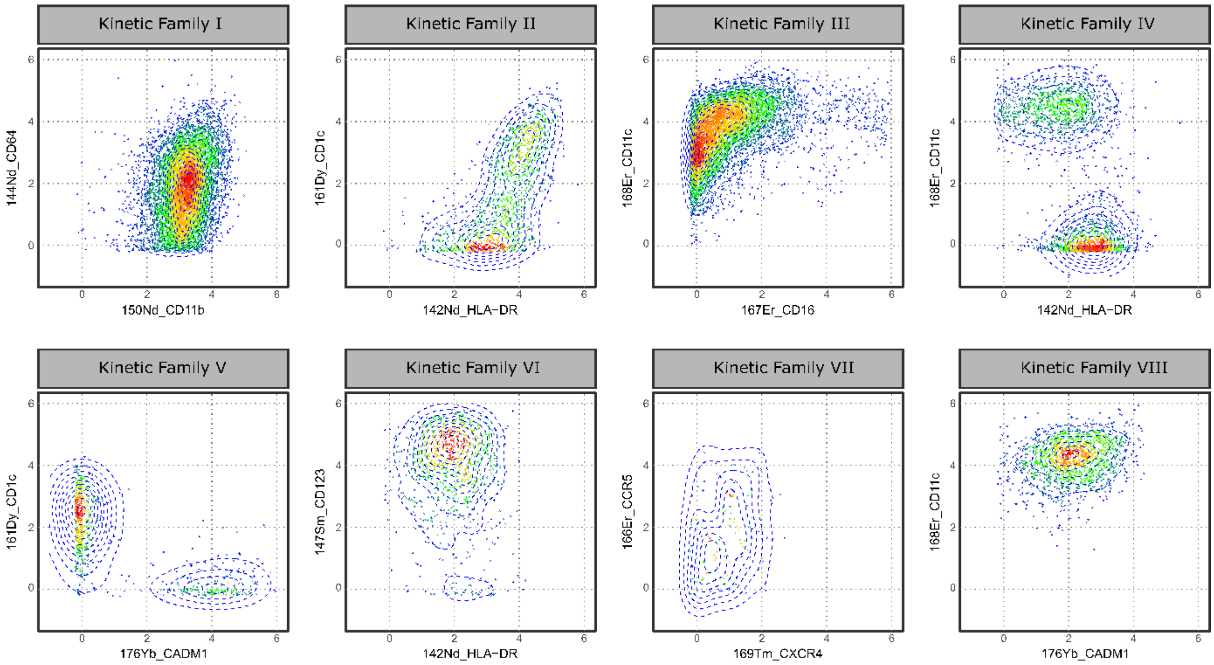

Figure S7: Main characteristics of kinetic families expression profiles (related to figure 5). Each dotplot represents the mean signal intensity (MSI) of all cells contained by a kinetic family (KF) without vaccines segregation. Markers are chosen according to the main population characteristics as defined in Table 1.

Table S1. **p-values of differences in cell numbers within phenotypic families (related to figure 4).** Comparisons were made using the Mann-Whitney Wilcoxon test. P-values ≤ 0.05 are shown in red.

	Conditions	Timepoints	PF1	PF3	PF19	PF10	PF8	PF15	PF2	PF22	PF25	PF12	PF23	PF21	PF20	PF9	PF24	PF11	PF27	PF13	PF28	PF17	PF5	PF4	PF26	PF16	PF14	PF6	PF18	PF7
Comparison between conditions	MPLA-SC vs SQ-IM	Baseline	0.94	0.70	0.59	0.82	0.82	0.82	0.70	0.94	0.82	0.09	0.18	0.18	0.09	0.48	0.39	**0.04**	0.31	0.18	0.94	0.82	1.00	0.70	0.48	0.39	1.00	0.94	0.70	0.82
		1st-24h	0.18	0.13	0.24	**0.04**	0.18	0.13	0.09	0.06	**0.03**	**0.03**	0.06	1.00	0.94	0.70	0.94	0.94	0.48	0.24	0.70	0.82	0.48	0.82	0.59	0.48	0.59	0.94	1.00	0.59
		2nd-0h	0.94	0.94	1.00	0.82	0.18	0.39	0.82	0.70	0.70	0.18	0.39	0.94	0.48	0.31	0.94	0.48	0.75	1.00	0.82	0.48	0.39	0.48	0.31	0.59	0.70	0.18	0.94	0.31
		2nd-24h	0.48	0.18	0.24	**0.02**	0.70	0.18	0.13	0.06	**0.02**	**0.03**	0.09	0.94	0.94	0.39	0.31	0.94	0.24	0.70	0.82	0.31	0.18	0.31	0.24	0.18	0.13	0.39	0.94	0.06
		3rd-0h	0.94	0.59	0.39	0.70	0.94	0.48	0.48	0.94	1.00	0.09	0.31	0.48	0.31	0.31	0.13	0.13	0.70	0.18	0.59	0.31	0.70	1.00	0.39	0.82	0.48	0.39	0.24	1.00
		3rd-24h	0.82	1.00	0.18	0.13	0.48	0.82	0.59	**0.03**	0.09	**0.00**	**0.002**	**0.03**	0.31	0.48	0.70	0.31	0.39	0.94	1.00	0.82	0.24	0.24	0.39	0.39	0.48	0.94	0.82	0.39
	SQ-IM vs MPLA-IM	Baseline	1.00	0.59	0.13	0.94	0.59	0.94	1.00	0.48	0.94	**0.03**	0.06	0.06	**0.03**	0.31	0.13	0.48	0.82	0.39	0.24	0.31	0.94	0.82	0.59	0.18	0.94	0.59	0.48	0.94
		1st-24h	0.18	0.06	0.24	**0.02**	0.18	0.06	0.24	**0.009**	**0.009**	**0.03**	0.31	0.39	0.24	0.13	0.09	0.48	0.18	0.13	0.94	0.48	1.00	0.94	0.94	0.94	0.48	0.94	1.00	0.82
		2nd-0h	0.48	0.39	0.82	0.39	0.39	1.00	0.82	0.94	0.70	0.94	1.00	1.00	0.94	0.31	0.31	0.59	0.13	0.70	0.59	0.31	0.31	0.70	0.82	0.82	0.24	0.24	0.48	0.24
		2nd-24h	0.09	**0.03**	**0.04**	0.06	0.06	**0.04**	**0.03**	0.06	**0.04**	0.59	0.48	1.00	0.70	0.82	0.59	1.00	0.06	0.09	0.31	0.09	**0.04**	0.39	0.24	0.18	0.09	0.31	0.31	0.13
		3rd-0h	0.06	0.06	0.06	**0.03**	0.09	0.18	0.59	0.06	0.09	0.39	0.59	0.59	0.48	0.48	0.94	0.82	0.09	0.59	0.59	0.13	**0.04**	0.09	0.13	0.24	0.06	0.31	0.24	0.09
		3rd-24h	0.39	0.31	1.00	0.13	0.31	0.94	0.59	0.59	0.59	0.94	0.48	0.94	0.70	0.48	**0.03**	0.39	0.18	1.00	0.70	1.00	1.00	0.94	0.18	0.59	0.09	0.70	0.94	0.09
	MPLA-IM vs MPLA-SC	Baseline	0.70	0.94	0.39	0.24	0.59	0.59	0.82	0.94	0.94	0.48	0.94	0.70	0.70	0.48	0.70	0.24	0.48	0.94	0.24	0.39	0.94	0.82	0.09	0.13	1.00	0.39	0.39	0.59
		1st-24h	0.82	0.94	0.94	0.48	1.00	1.00	0.59	0.48	0.82	0.94	0.94	0.13	0.31	**0.03**	0.09	0.70	0.48	1.00	0.70	0.70	0.48	0.94	0.39	0.48	0.70	0.59	0.94	1.00
		2nd-0h	0.31	0.13	0.94	0.39	**0.02**	0.24	0.59	0.48	0.39	0.18	0.18	0.94	0.59	0.82	0.24	0.39	0.59	0.59	0.82	0.94	0.70	0.70	0.24	0.82	0.48	0.94	0.39	0.94
		2nd-24h	0.18	0.31	0.31	0.09	0.09	0.06	0.18	0.09	0.09	0.13	0.59	0.82	0.94	0.39	0.82	0.70	0.48	0.31	0.59	0.59	0.39	0.82	0.82	0.59	0.39	0.70	0.59	0.48
		3rd-0h	0.24	**0.00**	0.09	0.09	**0.04**	0.13	1.00	0.24	0.13	0.06	0.59	0.70	0.31	0.59	0.94	0.06	0.48	0.18	0.48	0.13	0.18	0.48	0.48	0.82	0.18	0.18	0.24	0.31
		3rd-24h	**0.04**	0.06	0.39	0.09	**0.04**	0.06	0.59	0.94	0.82	0.31	0.13	0.06	0.48	0.82	0.70	0.70	0.70	0.39	0.31	0.18	0.70	0.31	0.82	0.94	0.18	0.31	0.24	0.24
Comparison with the previous timepoint	MPLA-IM	2nd-0h vs 1st-24h	0.22	0.09	0.31	0.84	0.09	0.22	0.06	0.09	0.06	**0.03**	0.31	**0.03**	**0.03**	**0.03**	0.09	0.31	0.31	0.06	0.09	0.16	**0.03**	**0.03**	0.84	0.22	1.00	0.44	0.22	0.56
		2nd-24h vs 2nd-0h	**0.03**	**0.03**	0.06	0.06	0.09	**0.03**	**0.03**	0.06	**0.03**	0.31	0.31	**0.03**	0.16	0.09	0.69	0.69	0.06	**0.03**	0.69	0.84	0.84	1.00	0.22	0.44	0.44	**0.03**	0.31	0.16
		3rd-0h vs 2nd-24h	**0.03**	**0.03**	0.22	0.06	0.22	0.06	0.06	0.06	0.06	0.69	0.69	**0.03**	0.09	0.06	0.31	1.00	0.69	1.00	**0.03**	0.22	1.00	0.69	0.06	0.16	0.44	**0.03**	0.31	**0.03**
		3rd-24h vs 3rd-0h	1.00	0.44	1.00	1.00	0.69	**0.03**	**0.03**	**0.03**	**0.03**	0.31	0.84	0.06	0.06	0.56	0.44	0.69	0.06	0.31	0.56	0.31	0.84	0.06	0.16	0.31	0.06	0.44	0.84	0.16
	MPLA-SC	2nd-0h vs 1st-24h	1.00	0.84	0.84	0.31	0.69	**0.03**	**0.03**	0.44	0.09	0.16	0.56	**0.03**	0.44	0.84	0.31	0.69	0.44	0.09	0.16	0.16	0.09	**0.03**	0.84	0.22	0.56	0.84	0.84	0.84
		2nd-24h vs 2nd-0h	0.16	0.16	0.84	**0.03**	0.16	**0.03**	0.16	**0.03**	**0.03**	0.69	1.00	0.56	0.56	0.31	0.22	1.00	0.31	0.06	0.44	0.22	0.22	0.22	1.00	0.84	1.00	0.16	0.44	0.31
		3rd-0h vs 2nd-24h	0.09	0.06	0.84	0.16	0.09	**0.03**	0.06	0.06	**0.03**	0.56	0.84	0.44	0.84	0.44	0.31	1.00	0.44	0.31	0.09	0.09	0.16	0.22	0.22	0.44	0.44	0.09	0.31	0.31
		3rd-24h vs 3rd-0h	0.22	0.44	0.84	1.00	0.09	**0.03**	**0.03**	**0.03**	**0.03**	**0.03**	1.00	0.09	0.84	1.00	0.31	1.00	0.09	0.16	1.00	0.69	1.00	**0.03**	0.16	0.16	0.06	0.84	0.56	0.06
	SQ-IM	2nd-0h vs 1st-24h	0.09	0.06	0.06	0.06	0.44	0.56	0.84	**0.03**	0.44	0.44	1.00	0.44	0.44	0.44	0.44	0.69	0.06	0.84	0.84	0.84	0.56	0.69	0.56	0.84	0.84	0.56	0.56	0.69
		2nd-24h vs 2nd-0h	0.84	0.84	0.16	0.31	0.69	0.84	0.56	0.44	0.56	0.56	0.09	0.06	0.16	**0.03**	**0.03**	0.22	0.44	0.84	0.06	0.16	0.16	0.16	0.31	0.06	0.06	0.16	0.44	0.22
		3rd-0h vs 2nd-24h	1.00	0.84	0.06	0.06	0.84	1.00	1.00	0.44	0.44	0.31	0.44	**0.03**	0.06	0.03	**0.03**	0.16	0.56	1.00	**0.03**	**0.03**	**0.03**	0.09	0.09	0.06	0.06	**0.03**	**0.03**	0.06
		3rd-24h vs 3rd-0h	0.09	1.00	0.16	**0.03**	0.06	0.31	**0.03**	0.31	0.22	0.69	0.31	0.16	0.69	0.84	0.44	0.69	0.22	0.69	1.00	0.16	0.44	1.00	1.00	0.84	0.69	0.56	0.44	1.00
Comparison with baseline	MPLA-IM	1st-24h	0.44	0.44	**0.03**	**0.03**	0.84	**0.03**	**0.03**	**0.03**	**0.03**	0.31	1.00	0.06	0.56	1.00	0.06	1.00	0.44	0.69	**0.03**	0.69	0.84	0.56	0.44	**0.03**	**0.03**	0.84	0.44	0.44
		2nd-0h	0.16	0.09	0.16	0.16	0.22	0.16	**0.03**	0.31	0.09	0.06	0.44	0.09	0.09	**0.03**	**0.03**	0.16	0.22	0.06	0.03	0.22	0.06	0.06	0.69	**0.03**	0.22	0.09	0.44	1.00
		2nd-24h	0.06	0.06	0.06	**0.03**	0.09	**0.03**	**0.03**	0.06	**0.03**	0.06	0.84	1.00	0.69	0.56	0.06	0.22	0.84	0.84	0.09	0.44	0.06	0.22	0.56	0.16	0.16	0.69	0.31	0.69
		3rd-0h	0.09	0.22	0.09	**0.03**	0.84	**0.03**	**0.03**	0.22	**0.03**	0.16	1.00	0.03	**0.03**	**0.03**	**0.03**	0.31	0.44	0.56	**0.03**	0.06	**0.03**	0.16	**0.03**	**0.03**	**0.03**	**0.03**	**0.03**	**0.03**
		3rd-24h	0.22	0.22	0.06	**0.03**	0.84	**0.03**	**0.03**	**0.03**	**0.03**	0.56	0.22	0.44	0.56	0.16	0.06	0.44	**0.03**	0.84	**0.03**	**0.03**	**0.03**	**0.03**	**0.03**	**0.03**	**0.03**	**0.03**	**0.03**	**0.03**
	MPLA-SC	1st-24h	1.00	1.00	0.31	0.31	1.00	**0.03**	**0.03**	0.06	**0.03**	0.31	0.56	0.56	1.00	0.22	**0.03**	0.84	1.00	0.84	0.44	0.44	0.16	1.00	0.56	1.00	1.00	0.31	0.31	0.84
		2nd-0h	0.56	0.84	0.84	0.84	0.56	0.16	0.06	0.09	0.16	0.84	0.31	0.44	0.69	0.09	0.16	0.84	0.31	**0.03**	0.44	0.84	0.09	**0.03**	0.56	0.69	1.00	0.56	0.56	0.44
		2nd-24h	0.44	0.16	0.69	**0.03**	0.16	**0.03**	**0.03**	**0.03**	**0.03**	1.00	0.56	1.00	1.00	0.44	0.06	0.44	0.09	0.44	0.44	0.31	0.44	0.22	0.16	1.00	0.84	0.22	0.44	0.56
		3rd-0h	0.84	0.84	0.31	0.44	0.84	0.09	**0.03**	**0.03**	0.06	0.69	0.44	0.16	0.84	0.44	0.06	1.00	0.84	0.06	1.00	0.56	0.44	0.16	0.44	0.56	1.00	1.00	0.69	0.84
		3rd-24h	0.22	0.31	0.44	0.31	0.16	**0.03**	**0.03**	**0.03**	**0.03**	0.16	**0.03**	0.16	0.56	0.22	**0.03**	1.00	0.31	0.44	0.69	1.00	0.09	**0.03**	0.84	0.09	0.16	1.00	1.00	0.31
	SQ-IM	1st-24h	**0.03**	**0.03**	**0.03**	0.06	0.06	0.09	**0.03**	0.84	0.22	0.22	0.16	0.03	0.16	0.44	0.84	**0.03**	0.56	0.31	0.03	0.16	1.00	0.69	0.31	0.56	0.31	0.16	0.06	0.06
		2nd-0h	0.84	0.69	0.69	1.00	0.56	0.22	0.06	0.06	0.16	0.84	0.16	0.69	0.69	0.44	0.31	1.00	0.16	0.56	1.00	0.69	0.31	0.44	0.22	0.84	1.00	0.56	0.09	0.16
		2nd-24h	0.84	0.84	0.16	0.44	0.31	0.03	**0.03**	0.69	0.69	1.00	0.31	0.09	0.16	0.22	0.09	**0.03**	0.16	0.56	0.06	**0.03**	0.09	0.22	0.22	0.56	0.09	**0.03**	0.06	0.06
		3rd-0h	1.00	0.84	0.31	0.16	0.84	0.09	0.06	0.09	0.06	0.31	0.06	0.44	0.31	**0.03**	**0.03**	0.56	0.69	0.84	0.22	0.22	0.06	0.22	0.31	0.44	0.56	0.31	1.00	0.44
		3rd-24h	0.09	0.31	0.69	0.69	0.31	**0.03**	**0.03**	0.06	**0.03**	1.00	0.84	0.22	1.00	0.16	0.09	0.44	0.84	0.16	0.31	1.00	0.44	0.31	1.00	0.22	0.56	1.00	0.84	0.84

Table S2. p-values of differences in cell number between kinetic families (related to figure 5). Comparisons were made using the Mann-Whitney Wilcoxon test. P-values ≤ 0.05 are shown in red.

Conditions		Timepoint	KF I	KF II	KF III	KF IV	KF V	KF VI	KF VII	KF VIII
Comparison between conditions	MPLA-SC vs SQ-IM	Baseline	0.937	0.240	0.937	0.937	0.132	0.132	0.180	0.589
		1st-24h	**0.041**	0.394	0.485	0.132	0.937	**0.009**	0.485	0.394
		2nd-0h	0.699	0.589	0.240	0.589	0.485	0.065	0.589	0.394
		2nd-24h	0.065	0.132	0.240	0.699	0.937	**0.026**	0.699	0.240
		3rd-0h	0.937	0.180	0.485	0.485	0.394	0.132	0.589	0.485
		3rd-24h	0.310	0.818	0.394	0.937	0.132	**0.004**	0.589	0.240
	MPLA-IM vs SQ-IM	Baseline	0.937	0.240	0.699	0.937	0.132	**0.015**	0.699	0.394
		1st-24h	**0.041**	1.000	0.937	0.485	1.000	**0.041**	0.093	0.699
		2nd-0h	0.589	0.699	0.485	0.310	0.818	0.937	0.394	0.699
		2nd-24h	**0.026**	0.065	0.132	**0.041**	1.000	0.485	0.065	0.180
		3rd-0h	0.485	0.310	0.394	0.699	0.818	1.000	0.394	0.310
		3rd-24h	0.065	0.699	0.065	0.093	0.818	0.589	0.180	0.180
	MPLA-IM vs MPLA-SC	Baseline	0.818	0.485	0.699	1.000	0.589	0.485	0.818	0.132
		1st-24h	0.937	0.240	1.000	0.589	0.699	0.699	0.589	0.589
		2nd-0h	0.132	0.937	0.818	0.093	0.937	0.093	0.937	0.394
		2nd-24h	0.093	0.240	0.485	0.132	0.818	0.132	0.310	0.699
		3rd-0h	**0.041**	0.394	0.310	0.093	0.818	0.132	0.240	0.937
		3rd-24h	0.132	0.699	0.310	0.093	0.132	0.240	0.589	1.000
Comparison with the previous timepoint	MPLA-IM	2nd-0h vs 1st-24h	0.313	0.688	1.000	0.156	**0.031**	0.063	0.156	1.000
		2nd-24h vs 2nd-0h	**0.031**	0.438	0.219	0.156	0.063	0.313	**0.031**	1.000
		3rd-0h vs 2nd-24h	0.063	0.563	0.156	0.688	**0.031**	1.000	0.844	**0.031**
		3rd-24h vs 3rd-0h	0.063	0.063	0.563	0.219	0.063	0.563	0.063	0.219
	MPLA-SC	2nd-0h vs 1st-24h	0.219	**0.031**	0.844	0.844	0.094	0.094	0.219	0.313
		2nd-24h vs 2nd-0h	**0.031**	0.844	0.219	0.438	0.688	1.000	0.313	1.000
		3rd-0h vs 2nd-24h	0.063	0.688	0.313	1.000	0.313	0.563	0.688	0.313
		3rd-24h vs 3rd-0h	0.063	0.063	0.094	0.313	0.094	0.063	0.156	0.094
	SQ-IM	2nd-0h vs 1st-24h	0.156	0.438	1.000	0.563	0.438	0.563	0.688	0.563
		2nd-24h vs 2nd-0h	1.000	0.063	0.156	0.438	0.063	0.438	0.688	0.094
		3rd-0h vs 2nd-24h	0.844	**0.031**	**0.031**	0.063	**0.031**	0.438	0.563	0.063
		3rd-24h vs 3rd-0h	0.563	0.844	0.688	0.156	0.563	1.000	0.313	0.844
Comparison with baseline	MPLA-IM	1st-24h	**0.031**	0.063	0.313	0.844	0.219	0.219	0.438	0.156
		2nd-0h	0.156	**0.031**	0.219	0.219	0.688	0.094	0.063	0.156
		2nd-24h	**0.031**	0.063	0.219	0.156	0.438	0.156	0.844	0.313
		3rd-0h	0.063	**0.031**	**0.031**	0.156	0.063	0.156	0.844	**0.031**
		3rd-24h	**0.031**	**0.031**	**0.031**	0.438	0.688	0.438	0.219	**0.031**
	MPLA-SC	1st-24h	0.219	**0.031**	0.688	0.844	0.844	0.313	1.000	1.000
		2nd-0h	1.000	0.156	0.688	0.844	0.563	1.000	0.094	1.000
		2nd-24h	0.094	0.156	0.438	0.844	0.844	1.000	0.563	0.563
		3rd-0h	0.688	0.438	0.844	0.844	0.313	0.688	0.313	0.844
		3rd-24h	0.219	**0.031**	0.313	0.563	0.219	0.063	0.438	0.156
	SQ-IM	1st-24h	0.063	0.563	0.063	0.063	**0.031**	0.219	0.313	0.438
		2nd-0h	0.688	0.438	0.563	1.000	0.844	0.313	0.438	0.563
		2nd-24h	1.000	0.563	0.063	0.313	0.063	0.844	0.313	0.219
		3rd-0h	0.313	0.063	0.688	0.219	0.844	0.156	1.000	0.844
		3rd-24h	1.000	0.063	1.000	0.688	0.438	1.000	0.438	0.844

Table S3. Myeloid panel used for the mass-cytometry experiment (related to STAR Methods section). The metal and antibody clone associations are shown. Intracellular markers are shown in bold.

Metal	Antibody	Clone
Pr(141)	CD66abce	TET2
Nd(142)	HLA-DR	L243
Nd(143)	CD3	SP34.2
Nd(144)	CD64	10.1
Nd(145)	CD8	RPAT8
**Nd(146)**	**IL6**	**MQ2.13A5**
Sm(147)	CD123	7G3
**Nd(148)**	**IL4**	**8D48**
Sm(149)	CD11a	HI111
Nd(150)	CD11b	ICRF144
Eu(151)	CD62L	SK11
Sm(152)	CD4	L200
Eu(153)	FceRI	AER37
Sm(154)	CD86	IT2.2
Gd(156)	CD172a	15-414 / REA144
**Gd(158)**	**IP10**	**6D4**
Tb(159)	CD45	D058-1283
**Gd(160)**	**IL1a**	**364/3B3**
Dy(161)	CD1c	AF-5910
**Dy(162)**	**IL-12**	**C8.6**
Dy(163)	CD32abc	FLI8.26
**Dy(164)**	**IFNa**	**LT27/295**
Ho(165)	CD39	eBioA1
Er(166)	CD195 (CCR5)	3A9
Er(167)	CD16	3G8
Er(168)	CD11c	3.9
Tm(169)	CD184 (CXCR4)	12G5
Er(170)	CD14	M5E2
**Yb(171)**	**IL8**	**6265.8**
Yb(172)	CD23	9P25
Yb(173)	CD141	1A4
Yb(174)	CD20	2H7
Lu(175)	CCR7	G043H7
Yb(176)	CADM1	3E 1

Pr: Praseodymium	Gd: Gadolinium	Er: Erbium
Nd: Neodymium	Tb: Terbium	Tm: Thulium
Sm: Samarium	Dy: Dysprosium	Yb: Ytterbium
Eu: Europium	Ho: Holmium	Lu: Lutecium
